# Modified “Allele-Specific qPCR” Method for SNP Genotyping Based on FRET

**DOI:** 10.3389/fpls.2021.747886

**Published:** 2022-01-10

**Authors:** Ruslan Kalendar, Akmaral Baidyussen, Dauren Serikbay, Lyudmila Zotova, Gulmira Khassanova, Marzhan Kuzbakova, Satyvaldy Jatayev, Yin-Gang Hu, Carly Schramm, Peter A. Anderson, Colin L. D. Jenkins, Kathleen L. Soole, Yuri Shavrukov

**Affiliations:** ^1^National Laboratory Astana, Nazarbayev University, Nur-Sultan, Kazakhstan; ^2^Institute of Biotechnology HiLIFE, University of Helsinki, Helsinki, Finland; ^3^Faculty of Agronomy, S. Seifullin Kazakh AgroTechnical University, Nur-Sultan, Kazakhstan; ^4^State Key Laboratory of Crop Stress Biology for Arid Areas, College of Agronomy, Northwest A&F University, Yangling, China; ^5^College of Science and Engineering, Biological Sciences, Flinders University, Adelaide, SA, Australia

**Keywords:** allele-specific primers, fluorescence and quenching, FRET-based method, genotyping, qPCR and plate reader instruments, single nucleotide polymorphism (SNP), universal probes

## Abstract

The proposed method is a modified and improved version of the existing “Allele-specific q-PCR” (ASQ) method for genotyping of single nucleotide polymorphism (SNP) based on fluorescence resonance energy transfer (FRET). This method is similar to frequently used techniques like Amplifluor and Kompetitive allele specific PCR (KASP), as well as others employing common universal probes (UPs) for SNP analyses. In the proposed ASQ method, the fluorophores and quencher are located in separate complementary oligonucleotides. The ASQ method is based on the simultaneous presence in PCR of the following two components: an allele-specific mixture (allele-specific and common primers) and a template-independent detector mixture that contains two or more (up to four) universal probes (UP-1 to 4) and a single universal quencher oligonucleotide (Uni-Q). The SNP site is positioned preferably at a penultimate base in each allele-specific primer, which increases the reaction specificity and allele discrimination. The proposed ASQ method is advanced in providing a very clear and effective measurement of the fluorescence emitted, with very low signal background-noise, and simple procedures convenient for customized modifications and adjustments. Importantly, this ASQ method is estimated as two- to ten-fold cheaper than Amplifluor and KASP, and much cheaper than all those methods that rely on dual-labeled probes without universal components, like TaqMan and Molecular Beacons. Results for SNP genotyping in the barley genes *HvSAP16* and *HvSAP8*, in which stress-associated proteins are controlled, are presented as proven and validated examples. This method is suitable for bi-allelic uniplex reactions but it can potentially be used for 3- or 4-allelic variants or different SNPs in a multiplex format in a range of applications including medical, forensic, or others involving SNP genotyping.

## Introduction

Single-nucleotide polymorphism (SNP) represents the smallest type of genetic difference in DNA between biological samples, where one nucleotide is replaced by another (Jehan and Lakhanpaul, 2006; Perkel, 2008; Jiang, 2013). Despite the simplicity of this change in the DNA, SNP analysis has emerged as one of the most powerful tools employed over a wide range of research from small-scale student-led investigations into specific SNPs, to high-throughput microarray technologies involving thousands of SNPs analyzed simultaneously (reviewed in Peatman, 2011; Thomson, 2014; Morgil et al., 2020; Zhu et al., 2021).

Many SNP genotyping platforms exist (reviewed in Kim and Misra, 2007; Schramm et al., 2019). Several use fluorescence resonance energy transfer (Didenko, 2001; Kaur et al., 2020a) as the basis for SNP visualization (Table 1). Such systems generally have two essential components: (1) allele-specific primers or molecular probe targeting the SNP and (2) a system of dye and quencher with at least one donor that produces a fluorescent signal and an acceptor that quenches the fluorescence when in close proximity (Chen and Sullivan, 2003; Giancola et al., 2006; Mamotte, 2006). In some methods, like Scorpions (Thelwell et al., 2000; Solinas et al., 2001) TaqMan (Schena et al., 2006; Jawhari et al., 2015) and Molecular Beacons (Hardinge and Murray, 2019), these two parts are combined in a single molecular probe.

**Table 1:** Main FRET-based methods of genotyping.

**Name**	**Allele-specific (AS) component**	**Components with fluorophore-dyes (F) and quenchers (Q)**	
		**Universal or not (whether separated from AS)**	**Probe shape, and positions of F and Q**
TaqMan	Targeting SNP is located in the middle of probe	Single probe joining AS and F/Q parts together	Linear shape. F and Q are in 5′- and 3′-ends, respectively
Molecular Beacon	As above	As above	“Hair-pin” shape. F and Q are in the ends
Amplifluor, STARP (STAR-PCR)	Two non-labeled AS forward primers with “tags” and one common reverse primer	Two universal probes with the same “tags”	“Hair-pin” shape. F and Q are in 5′-end and the middle of probe, respectively
rhAmp^a^	As above^a^	Possibly two universal probes^a^	Seems to be linear, where F and Q are located at the ends^a^
KASP^b^	As above^b^	Possibly four universal probes^b^	Seems to be four linear universal probes. F and Q are in 5′- and 3′-ends of separate probes, respectively^b^
AS-qPCR	As above	Two universal probes with dye-specific “tags” and corresponding barcode sequences, and two universal probes with quenchers and barcodes	All four universal probes linear. F and Q are in 5′- and 3′-ends of separate probes, respectively
Proposed advanced AS-qPCR	As above	Two universal probes with “tags” and dye-specific barcode sequence, and one common universal probe with quencher and barcode	All three universal probes are linear. F and Q are in 5′- and 3′-ends of separate probes, respectively

a *Information about rhAmp was taken from Supplementary Figure S1 presented on the website of Integrated DNA Technologies (https://sg.idtdna.com/pages/products/qpcr-and-pcr/genotyping/rhamp-snp-genotyping)*.

b *Information about KASP was taken from Supplementary Figure S1 presented on the website of LGC Genomics (http://info.biosearchtech.com/agrigenomics-pcr-based-kasp-genotyping?utm_campaign=SO-KASP-Targetedandutm_medium=cpcandutm_source=googleadsandgclid=EAIaIQobChMI1oXLhYWN8wIV-ZlmAh09qAEAEAAYASAAEgLJPvD_BwE)*.

*Both Figures are presented in Supplementary Material 1*.

In contrast, in other fluorescence resonance energy transfer (FRET)-based methods, such as Amplifluor, Kompetitive allele specific PCR (KASP), and semi-thermal asymmetric reverse PCR (STARP), these components are kept separate. That is, they have: (1) non-labeled allele-specific primers (ASPs); (2) universal probes (UPs) labeled with fluorophores and quencher (Nazarenko et al., 1997; Khripin, 2006). KASP, Amplifluor, and STARP produced a very strong and maybe even “revolutionary” impact on the further development of SNP technology. Simple and cheap allele-specific primers (ASPs) can be designed and ordered for each SNP separately, while the relatively expensive UPs with fluorophores and quenchers are ordered just once in a stock that can be used over a very long time in many different SNP target analyses. The principle of ASP-UP is similar to that of Molecular Beacons with the addition of specialized identical “tags” at the 5′-end of the ASP and the 3′-end of the UP (Myakishev et al., 2001).

In these methods, a set of non-labeled ASPs includes two forward primers, acting on a competitive basis, and a single common reverse primer. Additionally, one of two corresponding UPs is included either with or without “hair-pin” FRET structures ending with either FAM or HEX/VIC fluorophores. This approach allows for great flexibility in assay design, which translates into a higher overall success rate for SNP genotyping and detection of InDels (Insertion/Deletion). This principle is used in various methods, including commercially produced Amplifluor (Millipore-Thermo Fisher Scientific, MA, USA) and KASP markers (LGC Genomics, Teddington, UK) for a fluorescent signal generation that enable bi-allelic discrimination and genotyping of SNPs or InDels.

Amplifluor (Rickert et al., 2004; Fuhrman et al., 2008) and, particularly, KASP markers (Wang et al., 2015; Ryu et al., 2018, 2019; Udoh et al., 2020; Brusa et al., 2021) are very successful commercial products and currently popular over a range of research areas. However, KASP technology is relatively expensive, especially for small experiments (reviewed in Kaur et al., 2020b). Other ASP-UP methods, such as Amplifluor-like and similar, are less expensive since all methods are unrestricted and published (Rickert et al., 2004; Rasheed et al., 2017; Zotova et al., 2019, 2020). The recently reported STARP or STAR-PCR method is similar but with different sequences for the length and shape of the “stem-loop” in the “hair-pin” of their UPs (Rasheed et al., 2016; Long et al., 2017; Li et al., 2019; Wu et al., 2020). In contrast, another SNP genotyping method, “rhAmp,” is based on the use of H2-dependent RNase together with UPs and ASPs (Integrated DNA Technologies, USA). During amplification cycling, polymerase extension leads to degradation of the rhAmp probe, and a fluorescent signal is released (Dobosy et al., 2011; Massa et al., 2021).

Earlier, different FRET-based methods were compared including commercially produced reagents and Master-mixes, where allele discrimination showed similar levels of accuracy with AS-PCR, TaqMan, KASP, Amplifluor, and rhAmp SNP genotyping (Giancola et al., 2006; Rosas et al., 2014; Broccanello et al., 2018; Ayalew et al., 2019; Kadirvel et al., 2020). These reports give additional options for researchers to use their products and services.

In the original allele-specific quantitative PCR (ASQ) method (Lee et al., 2016), fluorophores and quenchers are on separate linear fragments with complementary sequences but without a “hair-pin” structure as used in Amplifluor (Rickert et al., 2004; Fuhrman et al., 2008). This original ASQ method was previously carried out only in medical research (Lee et al., 2016) and it was chosen as the “prototype” for the modified technique used in our present study on plant analysis.

The method we propose here is suitable for uniplex (and theoretically for multiplex) application and includes elements previously used in various approaches for SNP genotyping. The method requires two separate components: (1) *allele-specific part*; two or more AS primers target the SNP with identity in the penultimate positions of the 3′-end and specific tags in the 5′-end; (2) *universal part;* two or more universal probes (UPs) with corresponding tags and different fluorescent dyes in the 5′-end, and a single common universal probe with a quencher in the 3′-ends (Uni-Q), complementary to all UP tags.

Our proposed method can potentially be used for any type of polymorphic site target, with any SNP/InDel, as well as for multiplex PCR for different DNA targets or both cases simultaneously. The flexibility of the method is due to the universal part, which is the task-independent UPs with a single Uni-Q. Each UP can potentially be used for any task where differential and multiplex analysis of two or more targets is required.

The aim of this report was to demonstrate the functionality of the proposed ASQ method, the development of all components and conditions, and to show examples of their application for SNP genotyping in barley with comparisons to Amplifluor and KASP methods.

## Materials and Methods

### Computer Software and Primer Design

The FastPCR software with a computer-based calculator (https://primerdigital.com/fastpcr.html) was used for the design of ASQ assays and calculation of all parameters for ASPs and universal probes (UPs), including their common tags and fluorescence-specific parts (Kalendar et al., 2017; Kalendar, 2022). The melting temperature (T_m_) for primers and probes was calculated using FastPCR software, as indicated above, and presented in basic values. The T_m_ was calculated using a formula based on a Nearest neighbor thermodynamic theory with unified Gibbs free energy (dG), with entropy and enthalpy parameters (dS and dH, respectively) (Allawi and Santalucia, 1997; SantaLucia, 1998). Melting temperature was calculated for oligonucleotides at a concentration of 100-500 nM, depending on the specific oligonucleotide, and for standard PCR reaction buffer with 50 mM K^+^ and 3 mM Mg^2+^, or without magnesium for ASP sequences calculated without a tag. The FastPCR program was used to arrange the SNP preferably in the penultimate position at the 3′-end of the ASP. If no suitable penultimate position of the InDel site could be found, the program recommended an optimal variant of the design in one of three last nucleotides at 3′-end of ASP, which increases the reaction specificity and allele discrimination. InDels (Insertion-Deletion polymorphism) allow more options for automatic design of SNP site at the 3′-end of the ASP using the FastPCR program.

### Plant Material and DNA Extraction

Cultivated barley (*Hordeum vulgare* L.) was used, comprising the two feed varieties: (1) Natali. Origin: Russia (Catalog No: K-30957 at Vavilov Research Institute of Plant Industry, St.-Petersburg, Russia) (Zlotina et al., 2013) and (2) Auksiniai-2. Origin: Lithuania (Catalog EBDB ID: 36534 at ECPGR European Barley Database, Gatersleben, Germany; https://ebdb.ipk-gatersleben.de/apps/ebdb). Barley plants were grown in irrigated pots with soil in open natural conditions at the Nur-Sultan city Campus of KATU (S.Seifullin Kazakh AgroTechnical University), Nur-Sultan, Kazakhstan, in 2018 and 2019. At least 10–12 individual plants from each cultivar were used for DNA extraction but various numbers of biological replicates (from 3–4 to 10–12) were used in different tests for SNP genotyping, while 42 and 58 plants were used from their hybrids.

For PCR-fragment sequencing, one young, fully developed leaf was collected from each of the 1-month-old plants. Leaf samples were placed separately in 10 ml plastic tubes (not bulked), frozen, and kept at −20°C until DNA extraction. A phenol-chloroform method of DNA extraction was used as described earlier (Shavrukov et al., 2016; Zotova et al., 2018). The extracted DNA pellet was dissolved in 1/10 diluted TE buffer, and DNA concentration was measured with a NanoDrop spectrophotometer (Thermo Fisher Scientific, MA, USA), with quality assessed using 1 mg of DNA visualized after running in a 1% agarose gel. DNA samples were split in two, with one half used at Kazakh AgroTechnical University (KATU) and the other transported to Flinders University (Australia) for sequencing analysis.

For SNP genotyping, two pieces of the leaf at about 3 cm in length, were collected from individual plants as described above and placed in 1.1 ml collecting tubes in a 96-hole rack-box, frozen at −80°C for at least 2 h and used for freeze-dried DNA extraction following the original protocol (Shavrukov et al., 2010). The extracted DNA pellet was dissolved in 200 μl of sterile water. DNA concentration was measured with a NanoDrop spectrophotometer (Thermo Fisher Scientific, MA, USA), and adjusted to a concentration of 20 ng/μl for use in PCR with a known gene for quality control.

### Sequencing of the Target Gene in Barley

Genes *HvSAP16* (MLOC_52196 = AK360983) and *HvSAP8* (MLOC_43986 = AK372340), encoding stress-associated proteins in barley, were identified earlier (Baidyussen et al., 2020), and amplified PCR products were Sanger sequenced with subsequent capillary separation using the Applied Biosystems 3730/3730xl DNA Analyzer (Thermo Fisher Scientific, USA) for SNP identification in this study. Information about the primers used and sequences of the target gene, conditions of PCR, purification of PCR products, and sequencing is presented in Supplementary Material 2. The sequence fragment with the identified SNP in *HvSAP16* with designed ASPs is provided in the Results section. All information related to *HvSAP8* including sequences of the identified second SNP developed primers, which are T_m_ for both 3 mM Mg^2+^ and basic parameters, is presented in Supplementary Material 3.

### SNP Genotyping Comparison Using Three Different Methods: ASQ, Amplifluor-Like, and KASP

Experiments were carried out both in S. Seifullin Kazakh AgroTechnical University, Nur-Sultan (Kazakhstan), and Flinders University, Adelaide (Australia). Design of ASP and UP and PCR conditions for SNP genotyping in the proposed ASQ method are presented in the Results section, while the information for Amplifluor-like and KASP methods are provided in Supplementary Material 4. ASPs and UPs were obtained from “DNA Synthesis” (https://oligos.ru; Moscow, Russia) and Sigma-Aldrich (Sydney, Australia), respectively. KASP Master-mix was kindly provided by the University of Adelaide (Australia).

A QuantStudio-7 Real-Time PCR instrument (Thermo Fisher Scientific, MA, USA) and CFX96 Real-Time PCR Detection System (Bio-Rad, USA) were used in Kazakhstan and Australia, as described earlier (Khassanova et al., 2019; Sweetman et al., 2020). These instruments have detection systems with filters for FAM (Fluorescein) and VIC (2'-chloro-7'phenyl-1,4-dichloro-6-carboxy-fluorescein)/HEX (Hexachlorofluorescein) fluorophores and the conditions are described in the Results section. SNP identity calls were made automatically using software accompanying the instruments, but amplification curves were checked for each genotype manually for final allele discrimination. SNP genotyping experiments used at least three to eight biological replicates and were repeated three times (three technical replicates).

Identical microplate reader instruments, the FLUOstar Omega, Model 415-103 (BMG LabTech, Germany) were used at both S. Seifullin KATU and Flinders University, with FAM and HEX filters with absorption/emission ratio for 485/520 and 544/590 nm, respectively.

## Results

### Functional Principles of the ASQ Method—First Component: Allele-Specific Primers

**Figure 1:**
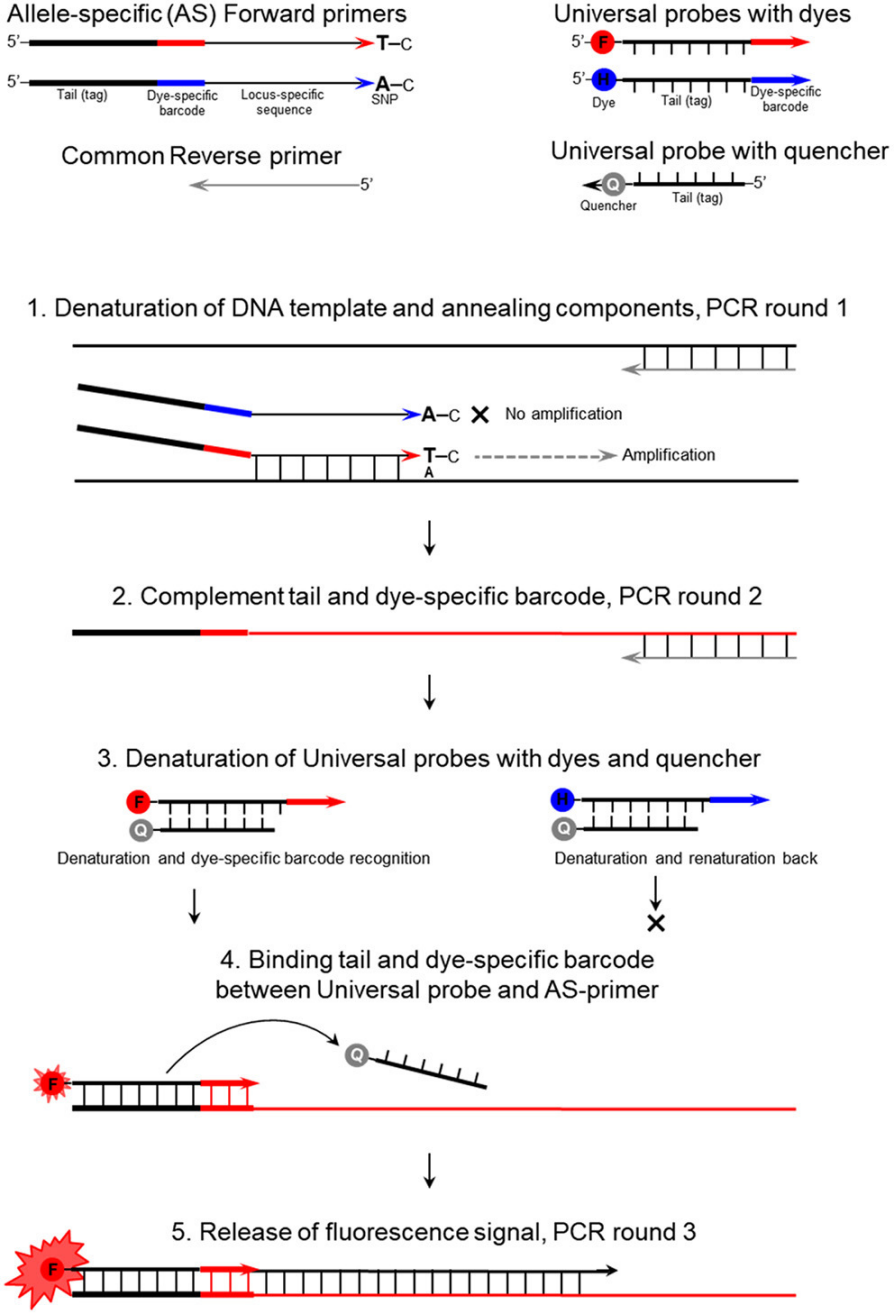
The mechanism of the ASQ assay in the example of a bi-allelic genotyping system. F, FAM; H, HEX; and Q, Quencher.

**Figure 2:**
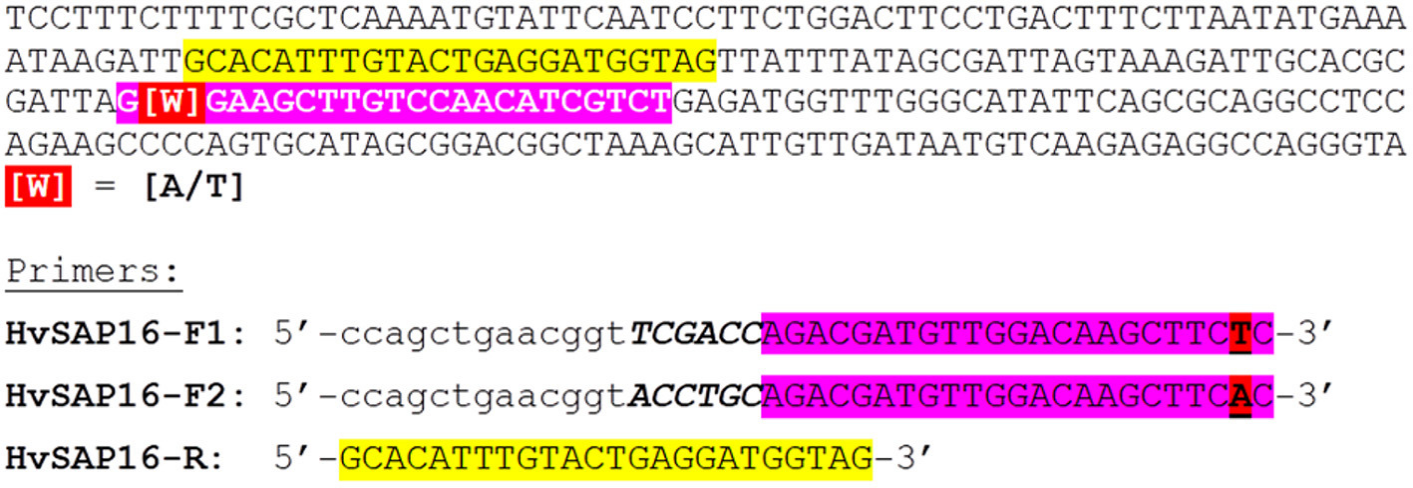
Example of fragment sequence in the promoter region of the *HvSAP16* gene of barley (*Hordeum vulgare* L.) with targeted SNP and designed ASPs for the proposed genotyping method. Forward and reverse primers are highlighted in pink and yellow, respectively. The SNP is designated as “[W]” in red and may be either “A” or “T” nucleotides. The design of two forward primers in the reverse-complement sequence is shown in the lower part of the Figure. The uncoloured 19-bp tag containing a 13-bp identical sequence (in lower case letters) and 6-bp unique “barcode” fragment (in Capital Italic letters) is shown. The common reverse primer (in yellow) is the same as present in the gene sequence.

The mechanism of the proposed ASQ assay is presented in Figure 1 and initially appears similar to those used in Amplifluor and KASP methods. The non-labeled ASPs are developed for each SNP under study and are separate from the second part of the method. The primer design for the *HvSAP16* gene of barley, with an amplicon size of 83-bp, is presented in Figure 2. ASPs consist of a set of three oligonucleotides: two forward primers and one common reverse primer, each around 22 bp in length, as usual for PCR primers (Table 2A). As in other methods, e.g., Amplifluor and KASP, the two forward primers target the alternative nucleotides at the SNP site and so act competitively.

**Table 2:** Allele-specific primers and universal probes with either fluorescent dyes or a quencher.

**Primer ID**	**Sequence (5^**′**^-3^**′**^)**	**T_**m**_ (**°**C)^**a**^**	**T_**m**_ (**°**C)^**b**^**	**dG (kcal /mole)**	
**(A) Allele-specific primers (ASPs)**					
HvSAP16-F1	ccagctgaacggt***TCGACC-***AGACGATGTTGGACAAGCTTCTC	63.5	55.7	−28.4	
HvSAP16-F2	ccagctgaacggt***ACCTGC-***AGACGATGTTGGACAAGCTTCAC	64.5	56.3	−28.7	
HvSAP16-R	GCACATTTGTACTGAGGATGGTAG	64.9	56.4	−28.2	
**Probe ID**	**Sequence (5** **′** **-3** **′** **)**	**T**_**m**_ **(°C)**^**c**^	**T**_**m**_ **(°C)**^**d**^	**dG (kcal/mole)**	**Fluorescent dye and quencher**
**(B) Universal probes (UPs)**					
UP-1	**FAM**-ccagctgaacggt***TCGACC***	66.5	58.2	−25.7	5′-FAM
UP-2	**HEX**-ccagctgaacggt***ACCTGC***	66.2	57.9	−25.5	5′-HEX, VIC or JOE
UP-3	**Cy3**-ccagctgaacggt***TGCAGG***	67.1	58.8	−25.9	5′-Cy3 or TAMRA
UP-4	**Cy5**-ccagctgaacggt***AGGTCG***	65.6	57.3	−25.3	5′-Cy5 or Liz
Uni-Q	*accgttcagctgg*-**Q**	53.7	46.1	−16.7	3′-Quencher (Eclipse, Dabsyl, BHQ1, TQ2 or BBQ-650)

***(A)** Allele-specific primers for the example of the HvSAP16 gene in barley which contains a common sequence (Upper case) but differs at the SNP position (Bold, underlined). A tag contains an identical 13-bp part (Lower case) followed by a 6-bp unique “barcode” fragment (Upper case, Italics)*.

***(B)** universal molecular probes are shown with four different fluorescent dyes (UP-1 to UP-4) and the single universal probe with a quencher (Uni-Q). Identical 13-bp parts of the tag and 6-bp unique barcode fragments are the same as in the ASPs. The universal molecular probe Uni-Q has a 13-bp tag in complement (Lower case, Italics)*.

a *Melting temperature (T_m_), calculated for ASPs, oligonucleotide concentration of 100 nM (500 nM for a common reverse primer oligonucleotide), 50 mM K^+^ with 3 mM Mg^2+^, for ASP sequences calculated without tag*.

b *T_m_ calculated for ASPs, oligonucleotide concentration of 100 nM (500 nM for a common reverse primer oligonucleotide), 50 mM K^+^, in the absence of Mg^2+^*.

c *T_m_ calculated for UPs, an oligonucleotide concentration of 300 nM (500 nM for Uni-Q oligonucleotide), 50 mM K^+^ with 3 mM Mg^2+^*.

d *T_m_ calculated for an oligonucleotide concentration of 300 nM (500 nM for Uni-Q oligonucleotide), 50 mM K^+^, in the absence of Mg^2+^*.

In our proposed method, the base in the ASPs that targets the SNP is placed penultimately at the 3′-end of each of two forward ASPs rather than at the terminal position as in other methods (Bui and Liu, 2009). Based on our prediction of the stereochemical outcome, the penultimate position in the 3′-end of the ASP provides greater allele discrimination.

The second difference is that the two forward primers have 19-bp tags added at the 5′-end. The first 13-bp from the 5′-end of the primer are identical in all forward ASPs, while a unique 6-bp “barcode” fragment is located the next, making the full length generally about 41-bp (22 + 19 bp). Therefore, the two forward ASPs differ from each other in the single nucleotide matching the SNP in the penultimate positions and the unique 6-bp barcode fragments. The two ASPs can be developed to either the forward or reverse strand of the target sequence (and this may also be done with KASP markers). The melting temperature of the ASP oligonucleotides with the complementary sequence is ~55–56°C (in the absence of Mg^2+^). The common reverse primer, which has no tag, is designed to be with a similar melting point to that of the forward primer and is positioned such that an amplicon of 50–100 bp (Table 2A).

### The Second Component: Universal Probes (UPs) With Individual Dyes or Quencher

The second universal part consists of fluorescently-labeled universal probes (UPs) (Table 2B). These oligonucleotides contain a common 19-bp tag identical to those in the 5′-end of the corresponding ASP tags, where the unique 6-bp barcode partly matches one of four corresponding UPs with different fluorophores (in this example, UP-1 and 2) (Table 2B). A minimum of two UPs are required in this case for bi-allelic SNP analysis in uniplex PCR, but up to three or four ASP-UP combinations potentially can be used for more complex SNP differences in the polymorphic position or using multiplex PCR for multiple target detection, subject to further experimental proof.

A single quencher oligonucleotide Uni-Q is complementary to the UPs' 5′-tags. Also, the quencher oligonucleotide is complementary to the 5′-proximal sequence in fluorescent probes (Uni-1 to Uni-4). The quencher oligonucleotide at the 3′-terminus carries a universal (meaning compatible with different dyes) wide-range quencher, that efficiently quenches various fluorescent dyes in the blue to red emission spectra, including, for example, Eclipse, Dabsyl, BBQ-650, BHQ1-2, or Tide Quencher 3. Differences in the tag sequences are determined by a short barcode 6-bp fragment that is not part of the universal tag sequence. The identical sequence of the 13-bp tag-fragment in each UP (UP-1 to 4) fully complements those in the Uni-Q (Table 2B).

Following the FRET principle, the fluorescent dye attached to the 5′-end of the UPs (UP-1 to 4) is located in close proximity to a quencher at the 3′-end of the Uni-Q probe due to the full complementarity of the 13-bp DNA-duplex. In this configuration, fluorescent emissions efficiently quench various fluorescent dyes in the emission spectra (Table 2B).

### Design of Tags and Barcode Sequences Specific for SNP Alleles in ASPs and Fluorescent Dye in UPs

For the design of the 6-bp barcode sequence, 348 highest-ranked at linguistic sequence complexity (LC) variants from 4,096 possible combinations of nucleotides were selected (Supplementary Material 5). There are several possible combinations and the most suitable 6-bp barcode sequences with similar thermodynamic characteristics were selected (Table 2B). The main criteria for the selection were based on (1) maximal differences for 4-bp between each possible pair combination of UPs (UP-1 to UP-4); (2) similar and as close as possible melting temperature (T_m_) and Gibbs free energy (dG) (Table 2B). Additionally, extra-6-bp barcode sequences provide at least 10°C higher T_m_ for all 19-bp UPs with fluorophores compared to 13-bp Uni-Q quencher sequences.

Uni-Q is designed in such a way that it does not inhibit the binding of UPs to the target amplification products. Therefore, the T_m_ for Uni-Q was significantly lower than all T_m_ values of all oligonucleotides used. At a T_m_ value of 60°C or higher, Uni-Q cannot bind to any of the UPs. This is a theoretical assumption based on the T_m_ values for Uni-Q and UPs. However, after the synthesis of the target product is completed, the detection of the product generated is performed by binding all free UPs with free Uni-Q.

### ASPs and UPs Have Similar Melting Temperatures but Differ in Annealing Access for Amplification

The ASPs targeting the SNP in the template and UPs with corresponding tags are designed to have a similar range of melting temperature (T_m_) between 55–56°C. However, ASPs will bind the SNP region of single-stranded target DNA after denaturation and in increasing numbers of the corresponding regions in PCR products following each amplification cycle. In contrast, UPs have a delay in their activity due to binding only PCR products with fragments complementary to the tags. As the PCR cocktail includes a mixture of both ASPs and UPs, their initial concentrations are very important for optimal genotyping results. In the case of equal concentrations, ASPs and UPs will have similar access in terms of their binding abilities to the amplified PCR products, which produces a low level of fluorescence signals. An even less effective process takes place with higher concentrations of ASPs than UPs, with minimal or no fluorescence.

The specificity of the binding of ASPs to the DNA target can be increased by lowing their concentration. Decreasing the concentration of the oligonucleotide reduces the T_m_ value of the oligonucleotide while increasing its effective binding could only be to the fully complementary site.

So, only lower ASPs and higher UPs, with a 2- to 3-fold difference in oligonucleotide concentration was successful. For example, in our optimal case, ASPs (both forward) and UPs (UP-1 and UP-2) had final concentrations of 0.1 and 0.3 μM, respectively. The use of a slightly higher annealing temperature of 60–62°C encourages preferential binding and amplification with UPs at the second round of PCR (**Table 4**). Consequently, a lower annealing temperature of 55°C with a higher concentration of UPs gives more opportunity to bind the 13-bp of single-stranded Uni-Q, but not the single-stranded amplified PCR product.

### Higher Concentration of Universal Probe With a Quencher (Uni-Q) Provides Better Fluorescence Background

Close interaction between the 5′-fluorophore and 3′-quencher occurs in the tags of UPs and Uni-Q. It is important that, in this situation, all UPs with fluorophores are bound by Uni-Q, and fluorescence is quenched completely. After progressive amplification and introgression of ASPs with specific tags to target amplicons, increasing numbers of corresponding UPs are bound and incorporated in the PCR product. At 55°C, all unused UPs renature and anneal with Uni-Q and so are quenched completely.

### Quencher Requirements

The common quencher of the Uni-Q must show broad-spectrum absorbance. This enables simultaneous quenching of multiple fluorophores with diverse spectra, from green FAM to red ROX used for uniplex, and all four dyes in multiplex PCR. The best choices may be Eclipse followed by Black Hole Quenchers (BHQ), which effectively quench a broad group of fluorescent dyes with emissions of 390–625 nm. Any available quencher could be used as a common universal quencher if it adequately quenches the fluorescence of all existing fluorophores. In the design of both Uni-Q and any of UP-1 to UP-4, the quencher and fluorophores are located in close proximity due to full complementary and hybridization. Additionally, the location of the quencher in the 3′-end of the Uni-Q oligonucleotide acts as an effective “stop-block” for Taq-polymerase and eliminates Uni-Q from primer-elongation.

### General Conditions, the Composition of PCR Cocktail Mix, and Optimized Level of Magnesium Mg^2+^

The PCR cocktail mix composition was 5 μl or 10 μl reaction volume, and the latter case is presented in Table 3. It is recommended that two pre-mixes are prepared, one for ASPs and the second for UPs but this can be re-calculated for each component separately. For ASPs, the final concentration of both forward ASPs should be about 0.1 μM, with the common reverse primer present in at least a 3- to 5-fold higher concentration (0.5 μM). The UPs pre-mix should be adjusted only once in preliminary tests, and aliquots of each component from 100 μM stock solutions can be mixed, used, wrapped in foil or dark-plastic tubes, and kept at −20°C for a very long time. The optimal concentration of UPs with fluorophores should be about 0.3 μM, and in a combination of two for bi-allelic SNP determination in uniplex format, while three or four UPs with different dyes can be used in multiplex format with three or four designated SNP alleles. The Uni-Q must be present in at least the same or double the concentration compared to the sum of all UPs containing 5′ fluorophores.

**Table 3:** The composition of PCR cocktail mix for the proposed ASQ method of SNP genotyping.

**Component**	**Concentration**	**Volume (μl)**	**Final concentration**
GoTaq Buffer (clear, without MgCl_2_)	5 ×	2.0	1 ×
MgCl_2_	25 mM	1.2	3.0 mM
dNTP	2 mM	1.0	0.2 mM
**ASPs pre-mix**			
ASP-F1	1 μM	1.0	0.1 μM
ASP-F2	1 μM		0.1 μM
ASP-R	5 μM		0.5 μM
**UPs pre-mix**			
UP-FAM:	3 μM	1.0	0.3 μM
UP-HEX/VIC	3 μM		0.3 μM
Uni-Q	6 μM		0.6 μM
Go-Taq polymerase	5 units/μl	0.08	0.04 units/μl
Sterile water	–	1.72	–
**Master mix**	–	**8.0**	–
DNA template	10 ng/μl	2.0	20 ng/μl
***Total***	–	**10.0**	–

**Figure 3:**
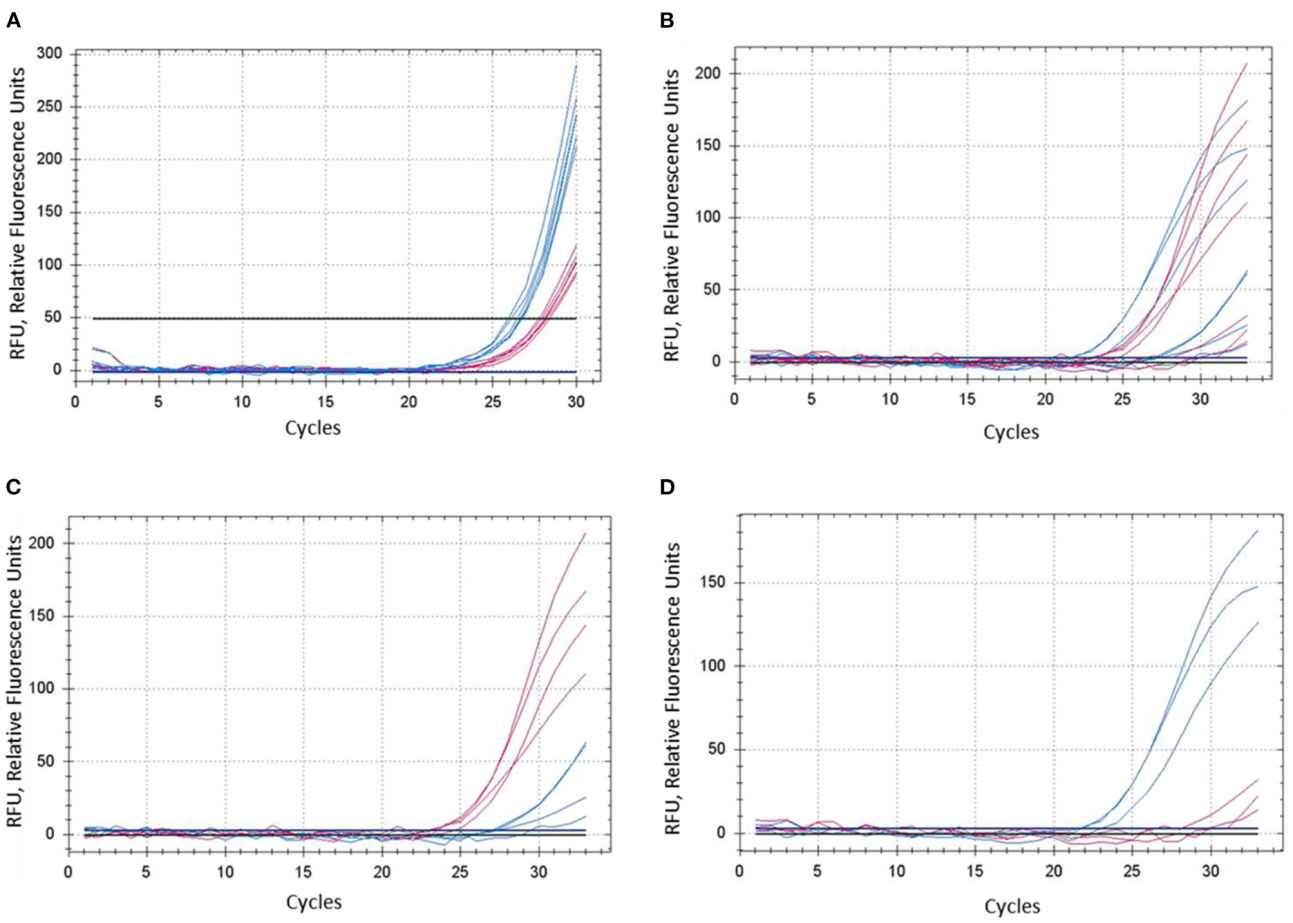
Comparison of dye fluorescence in the proposed ASQ method with different levels of Mg^2+^ in reactions. Concentration of MgCl_2_ was: **(A)** 1.5 mM and **(B**–**D)** 3.0 mM. In each test, shown in **(A)** and **(B)**, both homozygous genotypes “*aa*” and “*bb*” were present, seven plants in total. FAM- and HEX-amplification curves are designated by red and blue lines and correspond to alleles “*a*” and “*b*,” respectively. **(C,D)** Represent a sub-set of samples retrieved from **(B)**, where amplification curves are shown separately for four “*aa*” genotypes **(C)** and three “*bb*” genotypes **(D)**.

The concentration of magnesium Mg^2+^ is critical for optimal SNP genotyping using the proposed ASQ method. Best performance for most brands of standard Taq-polymerase requires a high Mg^2+^ concentration (3 mM), which is 1.7- to 2-fold higher than the concentration used in the Amplifluor-like method. However, at least one preliminary test is required, since lowering the optimal magnesium concentration will result in poor or non-specific amplification with incorrect allele discrimination (Figure 3A). Higher Mg^2+^ concentration will increase the efficiency of processive DNA Taq-polymerase but a magnesium concentration higher than optimal can lead to non-specific amplification.

A decrease in magnesium concentration reduces the efficiency of polymerase complexing with the DNA-duplex, which leads to a decrease in the amplification yield. Magnesium plays more of a role as a factor in the binding of the polymerase to the DNA-duplex than as a factor in PCR specificity. On amplification specificity, magnesium concentration within optimal limits (1.5–3 mM) makes no difference. As magnesium concentration decreases from 3 mM, PCR product formation shifts toward more distant PCR cycles. For this reason, we calculate T_m_ values for oligonucleotides with and without magnesium concentration. Because the presence of magnesium in solution has a diverse function in PCR, and the stability of the DNA-duplex in the presence of magnesium is more complex in nature than the presence of monovalent cations.

In contrast, the most favorable Mg^2+^ concentration shows much better differences in SNP genotyping (Figure 3B), where sub-sets of genotypes with different SNP alleles showed their clear and adequate amplification with subsequent correct allele discrimination (Figures 3C,D).

The remaining general components include a standard mixture of dNTPs and any suitable Taq-polymerase with its given buffer (Table 3). The brand and choice of Taq-polymerase is not critical and depends on the preferences of researchers and their own experiences in working with them. The suggested range of 0.04–0.05 units/μl of the enzyme in final concentration was optimal in our experiments. A lower concentration of Taq-polymerase can result in stronger background interference while too many Taq-polymerase results in rapid amplification with very narrow a window for clear and correct SNP allele discrimination. In general, a Hot-start Taq-polymerase usually required fewer cycles, but ultimately there seems to be little difference in SNP genotyping compared to regular Taq-polymerase.

Application of DMSO for PCR improvement is possible but it makes sense only in cases where no effective allele discrimination was found in regular conditions for SNP genotyping. However, it is important to note that the application of DMSO will affect all used oligonucleotides, including UPs with dyes and a quencher (Uni-Q), and it can result in a less effective quenching process. Therefore, added DMSO can be used in some cases.

Moving the SNP-site to the penultimate position significantly increases the differentiation of each allele, including moving the SNP-site to the third position from the 3′-end of the ASP. This is due to the stability and geometry of the hybridization DNA-duplex, and the nucleotide composition surrounding the SNP site. Depending on the geometry of the hybridization DNA-duplex, this complex can be either stable or not. Therefore, even in the penultimate position, SNP detection for some sequences will also not be effective. In this case, moving the SNP-site to the third position from the 3′-end of the ASP is required. The first 12 nucleotides from the 3′-end of the primers are critical for binding the primer to the DNA target. Therefore, within these 12 nucleotides, the SNP site from the 3′-end of the ASP can potentially be moved and preferably, the destabilization of the hybridization DNA-duplex should be maximal for each alternative allele close to the 3′-end of the ASP.

The recommended total volume of PCR for SNP genotyping using the modified ASP-UP method is from 5 to 10 μl per reaction in 384/96-well microplate formats, which yielded very good scoring results. A smaller volume of PCR with ASP-UP is more economical but requires greater accuracy in pipetting and manual operation, with a reasonable speed in loading PCR Master-mix and DNA template on ice to prevent evaporation. A robotic system of loading is obviously extremely accurate but also very expensive for small laboratories. In this regard, digital pipettes for loading Master-mix and multi-channel pipettes for the addition of templates can be very useful tools for accurate PCR preparation.

Template DNA extracted by any appropriate method with suitable quality control may be used for SNP genotyping if the Taq-polymerase is not inhibited by contaminants (Kalendar et al., 2021). DNA can be diluted and used in quite a wide range of concentrations from 5 to 50 nM, where 10 or 20 nM for 5 or 10 μl reactions, respectively, might be estimated as optimal for most plant species.

### Modified PCR Protocol for SNP Genotyping

The proposed PCR protocol for the modified ASQ method of SNP genotyping has some steps very similar to other FRET-based methods like Amplifluor or KASP, but there are important differences (Table 4). During the two rounds of PCR, the first one with 10 cycles had an annealing temperature of 55°C (Step 3) which allowed specific binding of ASPs to the template DNA in the region of the SNP. The extension temperature of 60–68°C (Step 4) is recommended for regular PCR. There were no differences found in experiments using “touch-down” annealing temperatures reduced by 0.6–1.0°C per cycle as recommended in the KASP method (Jatayev et al., 2017). Therefore, a fixed annealing temperature was used in the first round of amplification.

**Table 4:** PCR running protocol for genotyping using advanced ASQ method for SNP genotyping.

**Step**	**Temperature**	**Duration**	**Notes**
1	94°C	2 min	Initial denaturation. Can be variable depending on Taq-polymerase
2	94°C	10 s	First-round denaturation
3	55°C	20 s	First-round annealing
4	68°C	20 s	First-round extension
5			10 cycles repeat for steps 2–4
6	94°C	10 s	Second-round denaturation
7	62°C	20 s	Second-round annealing-extension
8	68°C	20 s	Second-round extension (optional)
9	55°C	30 s	Cooling, extension and Microplate read
10			30 cycles repeat for steps 6–9

More changes were made in the second round of PCR, where the annealing temperature of 62°C (Step 7) was optimal for binding of UPs to the target amplicons produced from the previous PCR round. This will make the PCR process more specific, particularly for binding of single-stranded amplified fragments with UPs and their corresponding fluorophores. The signal detection step takes place at Step 9, where the temperature was decreased from elongation (Step 8) at 68–55°C. The T_m_ value for Uni-Q is lower than all T_m_ values of all other oligonucleotides used. At T_m_ values of 60°C and higher, Uni-Q cannot bind to any of the UPs because it is in a totally free coil state.

After completion of amplicon synthesis (Step 8), Uni-Q effectively binds all free UPs at 55°C. At higher temperatures (Step 7), stable complexes between UPs and Uni-Q are not possible or poorly stable. Therefore, Uni-Q does not inhibit target amplification throughout amplification.

This additional annealing step provides a dramatic reduction of the second-step extension temperature by 13–17°C and will cause strong binding and renaturation of all amplified fragments and UPs with fluorophores. This temperature drop has an enormous impact on microplate reading results with dye fluorescence. The Uni-Q with quencher has a shorter fragment by 13 bp (Table 2) but its higher concentration (Table 3) presents the opportunity to bind all non-used UPs with fluorophores and quench all background fluorescence completely.

### Instruments for qPCR and End-Point Microplate Reader

The proposed ASQ method for SNP genotyping can be used with two types of equipment. Real-time qPCR instruments detect changes in fluorescence level after each cycle of amplification, which shows dynamic changes in a given dye or mixture of dyes. Amplification curves for fluorophores can be observed in real-time in each cycle. However, as expected, the detection of fluorescence is registered after at least 20–25 cycles depending on modifications and adjustments applied. The released fluorescence is directly related to several consequential processes, including the incorporation of ASPs in the binding of the SNP targeted region, amplification of the complementary PCR products, engagement of UPs with fluorophores, and finally detachment from Uni-Q and freedom from quenching. Comparison with a no-template control (sterile water, NTC) provides either manual or automatic normalization of Relative fluorescence units (RFU). Therefore, allele discrimination for dye fluorescence can be calculated at any cycle of the microplate reading. The presented SNP genotyping results will differ depending on the brand of qPCR instruments and associated computer software.

Alternatively, a simple “end-point” approach can be used with a PCR cycler and Microplate Reader with appropriate filters matching the wavelength of the fluorophores used. After completing the amplification protocol (Table 4), usually, in 96-well plate format, PCR products may be diluted and used directly for fluorescence measurements in the Microplate Reader. Allele discrimination can be simply calculated based on the ratio between the fluorescence of the dyes used, either manually or *via* the instrument software. An example of scoring results for FAM and HEX fluorescence units with end-point detection in a part of 96-well microplate with SNP genotyping using the proposed ASQ method is presented in Supplementary Material 6.

### Proposed ASQ Method for SNP Genotyping Analysis of Barley Gene *HvSAP16*

**Figure 4:**
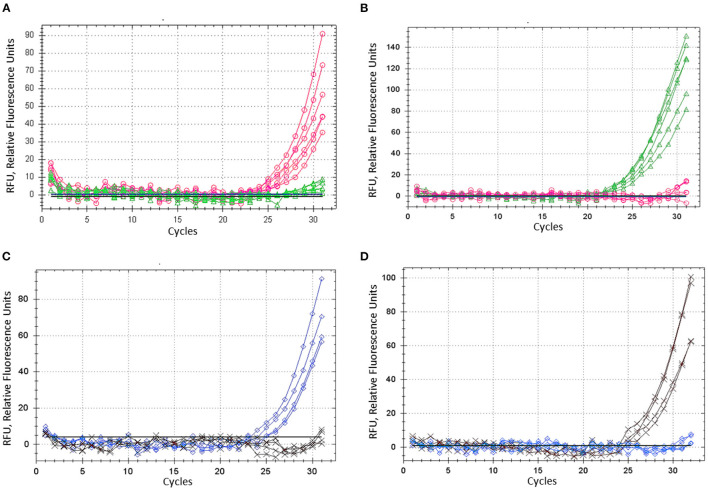
ASQ method optimisation: amplification of dye fluorescence depending on SNP allele, ASP, and corresponding UP in barley gene *HvSAP16* in two experiments. In the first test, six biological replicates were used and shown: **(A)** Allele 1—FAM (“A”) in plants cv. Natali with ASP-F1 and UP-1-FAM, shown as the red line and circles. **(B)** Allele 2—HEX (“T”) in plants cv. Auksiniai-2 with ASP-F2 and UP-2-HEX, shown as the green line and triangles. In the second test, four biological replicates were used and shown: **(C)** Allele 1—FAM (“A”) in plants cv. Natali with ASP-F1 and UP-1-FAM, shown as the blue line and rhombuses. **(D)** Allele 2—HEX (“T”) in plants cv. Auksiniai-2 with ASP-F2 and UP-2-HEX, shown as the brown line and crosses.

Barley plants from cvs. Natali and Auksiniai-2 with previously identified SNP alleles of the *HvSAP16* gene were used to confirm the accuracy of the modified ASQ method. This accuracy and specificity are based on the comparison of allele discrimination of known genotypes of the barley cultivars used, with SNP presence confirmed by Sanger sequencing. Several rounds with minor optimisation adjustments of the method were carried out, and amplification results for each of two fluorophores used (FAM and HEX) are shown for bi-allelic SNP identification in *HvSAP16* (Figure 4). RFU increased significantly in FAM and HEX after 24–25 and 22–23 cycles, respectively, in the amplification with studied barley genotypes. In each test, RFU of a single fluorescence only was recorded until about 30 cycles when RFU from the second fluorophore was practically negligible. However, even beyond 30 cycles, the difference in amplification of one fluorophore over the other was strong in all analyses, which reflects perfect discrimination of alleles in a wide range of “real-time” and “end-point,” after 25–33 cycles and beyond (Figure 4).

### Comparative Analysis of the *HvSAP16* Gene Using Amplifluor and KASP Methods and Validation of the ASQ Method Assessing SNP in *HvSAP8*

**Figure 5:**
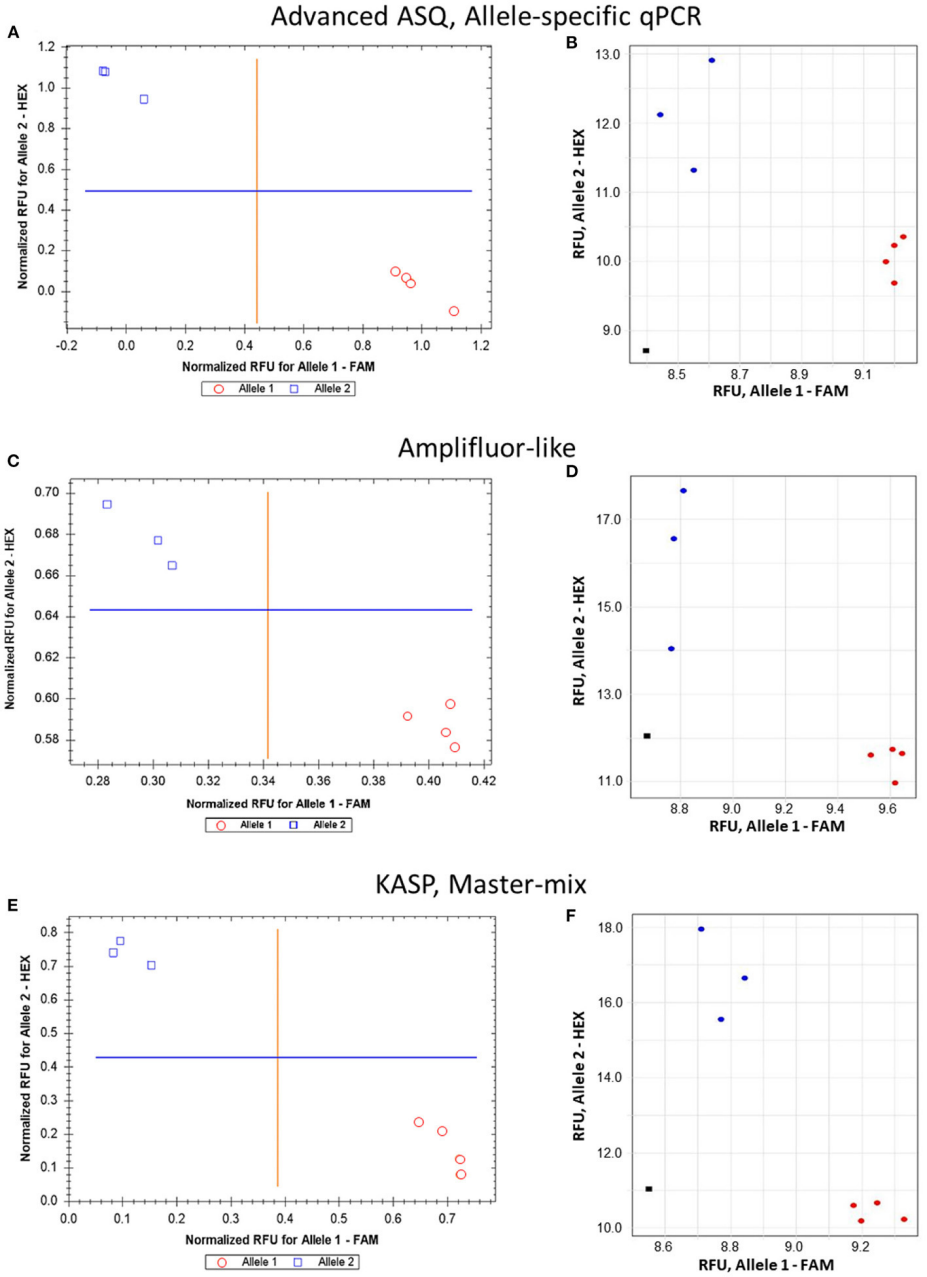
Comparison of allele discrimination between barley varieties using three different methods of SNP genotyping of the *HvSAP16* gene: **(A,B)** proposed ASQ method with ASPs and UPs as shown in Table 2; **(C,D)** Amplifluor-like method with ASPs and UPs as indicated in Material and Method section; **(E,F)** KASP Master-mix with UPs and Amplifluor-like ASP, as from **(C,D)** above. All three methods were checked in two types of qPCR instruments: Bio-Rad (left panels **A,C,E**) and Thermo Fisher Scientific (right panels **B,D,F**). SNP allele discrimination was based on Normalized reference fluorescence units (NRFU) in the Bio-Rad instrument and direct Reference fluorescence units (RFU) in the Thermo Fisher Scientific instrument. Allele 1 (FAM), cv. Natali is designated by red circles or red dots; and allele 2 (HEX), cv. Auksiniai-2 is shown in blue squares or blue dots. The normalization was made automatically by the qPCR software with the comparison of fluorescence data to no-template control (sterile water, NTC) shown by black squares in the Thermo Fisher Scientific instrument only. Four and three biological replicates were used for genotypes of Natali and Auksiniai-2, respectively.

The proposed ASQ method was compared with two other popular ones, Amplifluor-like and KASP. In addition, the same barley genotypes were tested using two different brands of qPCR instruments, Bio-Rad and Thermo Fisher Scientific, with their supplementary software analyses. As shown in Figure 5, similar accuracy and quite distinctive results were received for SNP allele discrimination with all three methods regardless of the qPCR instruments used.

**Figure 6:**
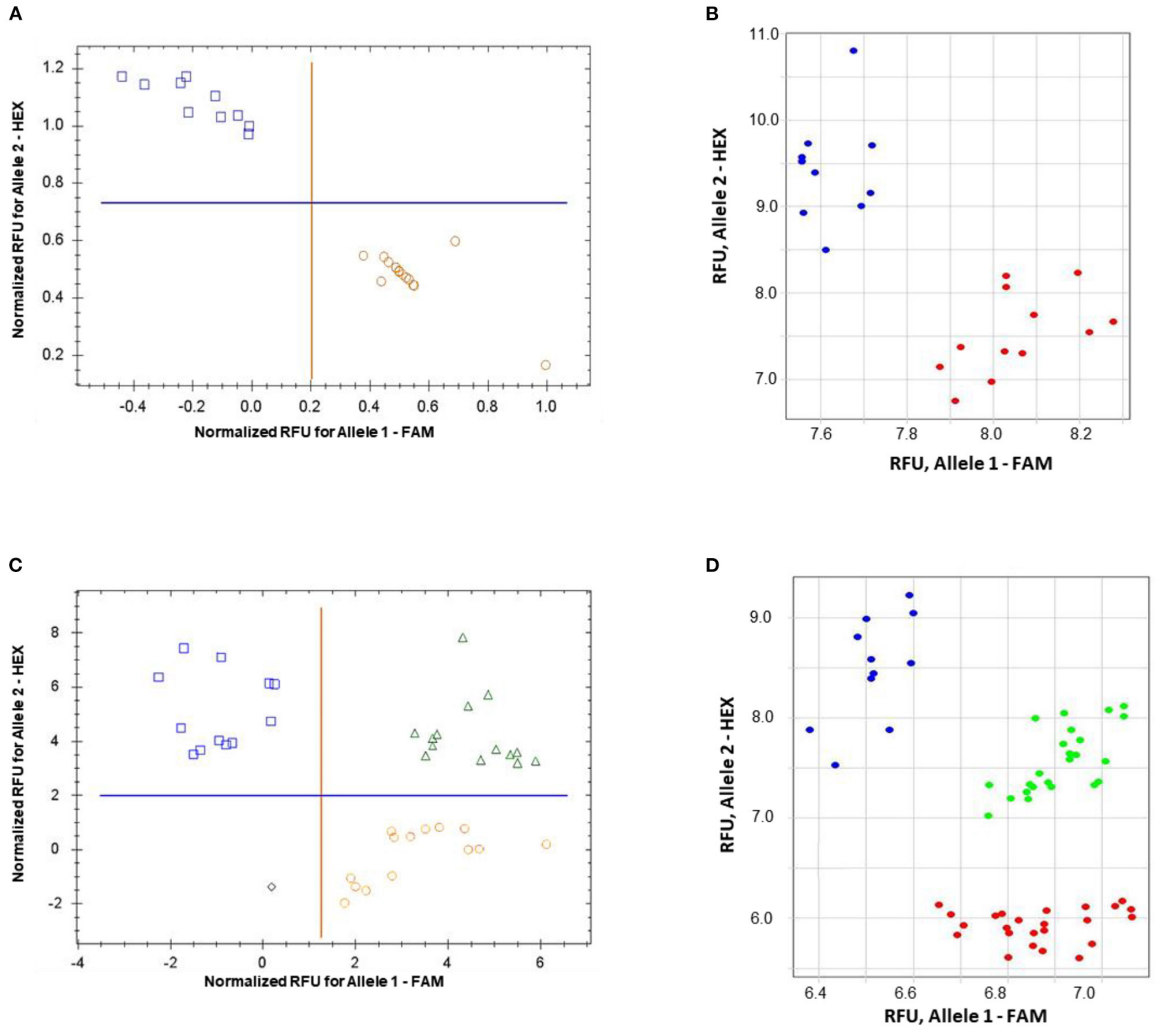
Allele discrimination for SNP genotyping of *HvSAP8* gene in barley varieties Natali and Auksiniai-2 **(A,B)**, and in the segregating populations [♀Natali × ♂Auksiniai-2] **(C,D)** using the proposed ASQ method in two types of qPCR instruments: Bio-Rad **(A,C)** and Thermo Fisher Scientific **(B,D)**. SNP allele discrimination was based on Normalized reference fluorescence units (NRFU) in the Bio-Rad instrument and direct Reference fluorescence units (RFU) in the Thermo Fisher Scientific instrument. Allele 1 (FAM), Natali haplotype is designated by red circles or red dots; and allele 2 (HEX), Auksiniai-2 haplotype is shown in blue squares or blue dots, heterozygotes are presented by green triangles or green dots. The normalization was made automatically by the qPCR software with the comparison of fluorescence data to no-template control (sterile water, NTC). Twelve and ten biological replicates were used for genotypes of Natali and Auksiniai-2, respectively **(A,B)**, while numbers of plants studied in the hybrid populations were *n* = 42 and *n* = 58, respectively **(C,D)**.

The validation of the proposed ASQ method was made using SNP in the barley *HvSAP8* gene. SNP allele discrimination for 12 and 10 biological replicates in Natali and Auksiniyai-2 genotypes, respectively, and for the segregating populations between these cultivars with *n* = 42 and *n* = 58, are presented in Figure 6. These results for SNP genotyping in both *HvSAP16* and *HvSAP8* genes in barley validated our conclusion that the proposed ASQ method is very accurate and effective for any SNP genotyping in barley, and we expect in other plant species and beyond.

## Discussion

Many SNP genotyping methods, especially TaqMan and Molecular Beacons, where allele-specific and dual-labeled parts are combined in a single molecular probe, are too expensive to be adopted by smaller laboratories (Edenberg and Liu, 2009; Kumar et al., 2012; You et al., 2018; Zhao et al., 2019; Zhu et al., 2021). The latest generation of SNP genotyping methods, for example, Amplifluor, KASP, rhAmp, and STARP, use universal molecular probes which are not related to any particular SNP and, therefore, can be applied to different SNP analyses (Rasheed et al., 2016; Long et al., 2017; Broccanello et al., 2018; Ayalew et al., 2019; Li et al., 2019; Wu et al., 2020). Such SNP technologies are significantly cheaper than the previous methods, but they still remain relatively expensive for laboratories with a limited budget.

The ASQ “prototype” (Lee et al., 2016) and the method proposed here had some differences compared to other methods with known UP structures. Although these methods use similar UPs, ASQ does not include a “hair-pin” loop. Moreover, the uncoupling of UPs into two independent units with a separate fluorophore and quencher in the ASQ method may give the appearance of a more complicated system but it results in a reduced cost per SNP, much greater flexibility, and easier adjustment of the method to any conditions.

More specifically, adding a single Uni-Q at 2- to 3-fold higher concentration and annealing at a lower temperature of 55°C (Step 8 of the amplification protocol, Table 4), ensures complete quenching of all non-incorporated UPs and thus a very low signal to noise ratio. Furthermore, the UPs and the Uni-Q differ in size (19- and 13-bp, respectively) and, therefore, have different annealing temperatures. This favors UP binding to amplified fragments and thus the generation of the signal.

The proposed ASQ method is based on a prototype that was used in medical research only (Lee et al., 2016) and it is unclear why it received so little attention outside the area. Our main modification of the method was the use of a single Uni-Q, which improves upon the original prototype and helps to address this oversight. In the proposed method, we completely redesigned the 19 bp tags, and their complements in the UPs were added to the ASPs. This resulted in a significant reduction in overall cost as only a single, universal quencher is then used. Although we did not show this experimentally, our modification with a single, common Uni-Q opens the way to assaying three or four allelic variants at a single SNP locus.

Although ASQ is similar to Amplifluor and KASP with regard to the use of two allele-specific forward primers, it differs in the positioning of the allele-specific nucleotide. In our protocol, the SNP is placed in the penultimate position rather than at the 3′ end. We believe this to be optimal because it allows for the use of proof-reading polymerases, which is important where template DNA quality may be poor.

Furthermore, the possibility of artificial mismatches added to the 5′-end of ASP in various methods can be considered (Liu et al., 2012; Choi et al., 2017; Han et al., 2017; Lu et al., 2020). Despite some possible increased discrimination between alleles (Hirotsu et al., 2010), the artificial mismatches in ASP can cause a reduction of total PCR efficiency, or inhibit the extension of the product by Taq polymerase (Ke and Wartell, 1993; Wu et al., 2009). Terminal mismatches can stabilize the DNA duplex structure, whereas internal mismatches can cause destabilization due to unfavorable helical constraints that prevent the formation of optimal base stacking and H-bond geometry (Stadhouders et al., 2010). Therefore, based on our estimations, artificial mismatches could improve allele discrimination but not in all cases.

There was an additional benefit of our protocol. Theoretically, the use of a single, common oligonucleotide to quench the fluorescence of all UPs could be extended for use in other protocols, such as quantitative and qualitative PCR. As an example, multiplex qPCR could be used to detect different targets simultaneously. Thus, for each individual target, the forward primer would contain a unique tag sequence for the corresponding UPs. The number of UPs could be expanded to equal the number of detection channels available on the device in any particular laboratory. The authors did not test this idea but suggested the potential extension of this method. An example of such applications would be the diagnosis of pathogens where simultaneous detection of multiple DNA targets and internal control was required. We can see no theoretical or practical difficulties in implementing this approach, including adapting to existing applications (Fors et al., 2000; Kreuzer et al., 2001; La Paz et al., 2007; Ragoussis, 2009; Gašparič et al., 2010; Löfström et al., 2015; Weck et al., 2021).

The presented results for the modified ASQ method are compared with those obtained from other methods. We estimated that our protocol was 2- to 10-fold cheaper than KASP or Amplifluor, based on cost per SNP (Supplementary Material 7), and even cheaper than other methods. The synthesis and purification of dual- or single-labeled fluorogenic oligonucleotides were the most expensive part of all methods. The use of Uni-Q with a common quencher in our method allows a significant reduction in overall cost. This improvement made our modified ASQ genotyping method a more adaptable, universal, cost-effective, and high-throughput genotyping alternative, and thus applicable to laboratories with a limited budget for the cost of SNP genotyping.

## Data Availability Statement

The original contributions presented in the study are included in the article/Supplementary Materials, further inquiries can be directed to the corresponding authors.

## Author Contributions

RK conceived the idea and provided bioinformatics analyses to design primers, tags and barcode sequences. AB, DS, LZ, GK, and MK generated results in Kazakhstan and carried out data analysis. SJ, Y-GH, and KS supervised the project and provided financial support for the research. CS, PAA, and CLDJ helped with illustrations, previous publications and the manuscript editing; YS coordinated all groups and prepared initial draft of the manuscript. All authors reviewed and approved the final manuscript.

## Funding

This work was supported by the Science Committee of the Ministry of Education and Science and Ministry of Agriculture of the Republic of Kazakhstan (AP08855353 for RK; BR10765000 for GK and MK) in the framework of program funding for research; the Key Research and Development Program of Shaanxi, China (Program No. 2021KWZ-23 for Y-GH). This study was also partially funded by Primer Digital Ltd (https://primerdigital.com). Open access funding was provided by the University of Helsinki (Finland), including Helsinki University Library, *via* RK. All funders provided financial support for the research but did not have any additional role in the study design, data collection and analysis, decision to publish, or preparation of the manuscript.

## Conflict of Interest

RK is employed by PrimerDigital Ltd. The remaining authors declare that the research was conducted in the absence of any commercial or financial relationships that could be construed as a potential conflict of interest.

## Publisher's Note

All claims expressed in this article are solely those of the authors and do not necessarily represent those of their affiliated organizations, or those of the publisher, the editors and the reviewers. Any product that may be evaluated in this article, or claim that may be made by its manufacturer, is not guaranteed or endorsed by the publisher.

## References

[B1] Allawi H. T. Santalucia J. J. (1997). Thermodynamics and NMR of internal G-T mismatches in DNA. Biochemistry 36, 10581–10594. 10.1021/bi962590c 9265640

[B2] Ayalew H. Tsang P. W. Chu C. Wang J. Liu S. Chen C. . (2019). Comparison of TaqMan, KASP and rhAmp SNP genotyping platforms in hexaploid wheat. PLoS ONE 14:e0217222. 10.1371/journal.pone.0217222 31116793 PMC6530864

[B3] Baidyussen A. Aldammas M. Kurishbayev A. Myrzabaeva M. Zhubatkanov A. Sereda G. . (2020). Identification, gene expression and genetic polymorphism of zinc finger A20/AN1 stress-associated genes, *HvSAP*, in salt stressed barley from Kazakhstan. BMC Plant Biol. 20:156. 10.1186/s12870-020-02332-4 33050881 PMC7556924

[B4] Broccanello C. Chiodi C. Funk A. McGrath J. M. Panella L. Stevanato P. (2018). Comparison of three PCR-based assays for SNP genotyping in plants. Plant Methods 14:28. 10.1186/s13007-018-0295-6 29610576 PMC5872507

[B5] Brusa A. Patterson E. L. Gaines T. A. Dorn K. Westra P. Sparks C. D. . (2021). A needle in a seedstack: an improved method for detection of rare alleles in bulk seed testing through KASP. Pest Manag. Sci. 77, 2477–2484. 10.1002/ps.6278 33442897

[B6] Bui M. Liu Z. (2009). Simple allele-discriminating PCR for cost-effective and rapid genotyping and mapping. Plant Methods 5:1. 10.1186/1746-4811-5-1 19133153 PMC2648961

[B7] Chen X. Sullivan P. F. (2003). Single nucleotide polymorphism genotyping: biochemistry, protocol, cost and throughput. Pharmacogenomics J. 3, 77–96. 10.1038/sj.tpj.6500167 12746733

[B8] Choi S. J. Ramekar R. V. Kim Y. B. Kim S. W. Noh H. S. Lee J. K. . (2017). Molecular authentication of two medicinal plants *Ligularia fischeri* and *Ligularia stenocephala* using allele-specific PCR (AS-PCR) strategy. Genes Genomics 39, 913–920. 10.1007/s13258-017-0554-3

[B9] Didenko V. V.. (2001). DNA probes using fluorescence resonance energy transfer (FRET): Designs and applications. BioTechniques 31, 1106–1121. 10.2144/01315rv02 11730017 PMC1941713

[B10] Dobosy J. R. Rose S. D. Beltz K. R. Rupp S. M. Powers K. M. Behlke M. A. . (2011). RNase H-dependent PCR (rhPCR): improved specificity and single nucleotide polymorphism detection using blocked cleavable primers. BMC Biotechnol. 11:80. 10.1186/1472-6750-11-80 21831278 PMC3224242

[B11] Edenberg H. J. Liu Y. (2009). Laboratory methods for high-throughput genotyping. Cold Spring Harb. Protoc. 4, 183–193. 10.1101/pdb.top62 20150074

[B12] Fors L. Lieder K. W. Vavra S. H. Kwiatkowski R. W. (2000). Large-scale SNP scoring from unamplified genomic DNA. Pharmacogenomics 1, 219–229. 10.1517/14622416.1.2.219 11256593

[B13] Fuhrman L. E. Shianna K. V. Aballay A. (2008). High-throughput isolation and mapping of *C. elegans* mutants susceptible to pathogen infection. PLoS ONE 3:e2882. 10.1371/journal.pone.0002882 18682730 PMC2478710

[B14] Gašparič M. B. Tengs T. La Paz J. L. Holst-Jensen A. Pla M. Esteve T. . (2010). Comparison of nine different real-time PCR chemistries for qualitative and quantitative applications in GMO detection. Anal. Bioanal. Chem. 396, 2023–2029. 10.1007/s00216-009-3418-0 20087729

[B15] Giancola S. McKhann H. I. Bérard A. Camilleri C. Durand S. Libeau P. . (2006). Utilization of the three high-throughput SNP genotyping methods, the GOOD assay, Amplifluor and TaqMan, in diploid and polyploid plants. Theor. Appl. Genet. 112, 1115–1124. 10.1007/s00122-006-0213-6 16453133

[B16] Han E. H. Lee S. J. Kim M. B. Shin Y. W. Kim Y. H. Lee S. W. (2017). Molecular marker analysis of *Cynanchum wilfordii* and *C. auriculatum* using the simple ARMS-PCR method with mismatched primers. Plant Biotechnol. Rep. 11, 127–133. 10.1007/s11816-017-0432-0

[B17] Hardinge P. Murray J. A. H. (2019). Lack of specificity associated with using molecular beacons in loop mediated amplification assays. BMC Biotechnol. 19:55. 10.1186/s12896-019-0549-z 31370820 PMC6676609

[B18] Hirotsu N. Murakami N. Kashiwagi T. Ujiie K. Ishimaru K. (2010). Protocol: A simple gel-free method for SNP genotyping using allele-specific primers in rice and other plant species. Plant Methods 6:12. 10.1186/1746-4811-6-12 20409329 PMC2876155

[B19] Jatayev S. Kurishbaev A. Zotova L. Khasanova G. Serikbay D. Zhubatkanov A. . (2017). Advantages of Amplifluor-like SNP markers over KASP in plant genotyping. BMC Plant Biol. 17:254. 10.1186/s12870-017-1197-x 29297326 PMC5751575

[B20] Jawhari M. Abrahamian P. Sater A. A. Sobh H. Tawidian P. Abou-Jawdah Y. (2015). Specific PCR and real-time PCR assays for detection and quantitation of “*Candidatus* Phytoplasma phoenicium.” Mol. Cell. Probes 29, 63–70. 10.1016/j.mcp.2014.12.003 25543009

[B21] Jehan T. Lakhanpaul S. (2006). Single nucleotide polymorphism (SNP)—methods and applications in plant genetics: a review. Indian J. Biotechnol. 5, 435–459. http://hdl.handle.net/123456789/5608

[B22] Jiang G. L.. (2013). Molecular markers and marker-assisted breeding in plants, in Plant Breeding from Laboratories to Fields, ed Andersen S. B. (London: InTech), 45–83. 10.5772/52583

[B23] Kadirvel P. Veerraju C. Senthilvel S. Yadav P. Kiran B. U. Shaik M. . (2020). Marker-assisted selection for fast-track breeding of high oleic lines in safflower (*Carthamus tinctorious* L.). Ind. Crops Prod. 158:112983. 10.1016/j.indcrop.2020.112983

[B24] Kalendar R.. (2022). A guide to using FASTPCR software for PCR, *in silico* PCR, and oligonucleotide analysis. in PCR Primer Design. Methods in Molecular Biology, Vol. 2392, ed Basu C. (New York: Humana), 223–43. 10.1007/978-1-0716-1799-1_16 34773626

[B25] Kalendar R. Boronnikova S. Seppanen M. (2021). Isolation and purification of DNA from complicated biological samples, in Molecular Plant Taxonomy. Methods in Molecular Biology, Vol. 2222, ed Besse P.. (New York: Humana), 57-67. 10.1007/978-1-0716-0997-2_3 33301087

[B26] Kalendar R. Khassenov B. Ramanculov E. Samuilova O. Ivanov K. I. (2017). FastPCR: an *in silico* tool for fast primer and probe design and advanced sequence analysis. Genomics 109, 312–319. 10.1016/j.ygeno.2017.05.005 28502701

[B27] Kaur A. Kaur P. Ahuja S. (2020a). Förster resonance energy transfer (FRET) and applications thereof. Anal. Methods 12, 5532–5550. 10.1039/d0ay01961e 33210685

[B28] Kaur B. Mavi G. S. Gill M. S. Saini D. K. (2020b). Utilization of KASP technology for wheat improvement. Cereal Res. Commun. 48, 409–421. 10.1007/s42976-020-00057-6

[B29] Ke S. H. Wartell R. M. (1993). Influence of nearest neighbor sequence on the stability of base pair mismatches in long DNA: determination by temperature-gradient gel electrophoresis. Nucleic Acids Res. 21, 5137–5143. 10.1093/nar/21.22.5137 8255768 PMC310628

[B30] Khassanova G. Kurishbayev A. Jatayev S. Zhubatkanov A. Zhumalin A. Turbekova A. . (2019). Intracellular vesicle trafficking genes, *RabC*-GTP, are highly expressed under salinity and rapid dehydration but down-regulated by drought in leaves of chickpea (*Cicer arietinum* L.). Front. Genet. 10:40. 10.3389/fgene.2019.00040 30792734 PMC6374294

[B31] Khripin Y.. (2006). High-throughput genotyping with energy transfer-labeled primers, in Fluorescent Energy Transfer Nucleic Acid Probes: Designs and Protocols. Methods in Molecular Biology, Vol. 335. ed Didenko V. V. (Totowa: Humana Press), 215–240. 10.1385/1-59745-069-3:215 16785631

[B32] Kim S. Misra A. (2007). SNP genotyping: technologies and biomedical applications. Annu. Rev. Biomed. Eng. 9, 289–320. 10.1146/annurev.bioeng.9.060906.152037 17391067

[B33] Kreuzer K. A. Bohn A. Lupberger J. Solassol J. Le Coutre P. Schmidt C. A. (2001). Simultaneous absolute quantification of target and control templates by real-time fluorescence reverse transcription-PCR using 4-(4′-dimethylaminophenylazo)benzoic acid as a dark quencher dye. Clin. Chem. 47, 486–490. 10.1093/clinchem/47.3.486 11238301

[B34] Kumar S. Banks T. W. Cloutier S. (2012). SNP discovery through next-generation sequencing and its applications. Int. J. Plant Genomics 2012:831460. 10.1155/2012/831460 23227038 PMC3512287

[B35] La Paz J. L. Esteve T. Pla M. (2007). Comparison of real-time PCR detection chemistries and cycling modes using Mon810 event-specific assays as model. J. Agric. Food Chem. 55, 4312–4318. 10.1021/jf063725g 17488028

[B36] Lee H. B. Schwab T. L. Koleilat A. Ata H. Daby C. L. Cervera R. L. . (2016). Allele-specific quantitative PCR for accurate, rapid, and cost-effective genotyping. Hum. Gene Ther. 27, 425–435. 10.1089/hum.2016.011 26986823 PMC4931339

[B37] Li P. Su T. Wang H. Zhao X. Wang W. Yu Y. . (2019). Development of a core set of KASP markers for assaying genetic diversity in *Brassica rapa* subsp. chinensis Makino. Plant Breed. 138, 309–324. 10.1111/pbr.12686

[B38] Liu J. Huang S. Sun M. Liu S. Liu Y. Wang W. . (2012). An improved allele-specific PCR primer design method for SNP marker analysis and its application. Plant Methods 8:34. 10.1186/1746-4811-8-34 22920499 PMC3495711

[B39] Löfström C. Josefsen M. H. Hansen T. Søndergaard M. S. R. Hoorfar J. (2015). Fluorescence-based real-time quantitative polymerase chain reaction (qPCR) technologies for high throughput screening of pathogens. in High Throughput Screening for Food Safety Assessment. eds Bhunia A. K. Kim M. S. Taitt C. R. (Amsterdam: Elsevier), 219–248.

[B40] Long Y. M. Chao W. S. Ma G. J. Xu S. S. Qi L. L. (2017). An innovative SNP genotyping method adapting to multiple platforms and throughputs. Theor. Appl. Genet. 130, 597–607. 10.1007/s00122-016-2838-4 27942775

[B41] Lu J. Hou J. Ouyang Y. Luo H. Zhao J. Mao C. . (2020). A direct PCR-based SNP marker-assisted selection system (D-MAS) for different crops. Mol. Breed. 40:9. 10.1007/s11032-019-1091-3

[B42] Mamotte C. D.. (2006). Genotyping of single nucleotide substitutions. Clin. Biochem. Rev. 27, 63–75. https://www.ncbi.nlm.nih.gov/labs/pmc/articles/PMC1390795 16886048 PMC1390795

[B43] Massa A. N. Bressano M. Soave J. H. Buteler M. I. Seijo G. Sobolev V. S. . (2021). Genotyping tools and resources to assess peanut germplasm: smut-resistant landraces as a case study. PeerJ 9:e10581. 10.7717/peerj.10581 33575123 PMC7849506

[B44] Morgil H. Gercek Y. C. Tulum I. (2020). Single nucleotide polymorphisms (SNPs) in plant genetics and breeding, in The Recent Topics in Genetic Polymorphisms, ed Çalişkan M. (London: InTech Open), 825–400. 10.5772/intechopen.91886

[B45] Myakishev M. V. Khripin Y. Hu S. Hamer D. H. (2001). High-throughput SNP genotyping by allele-specific PCR with universal energy-transfer-labeled primers. Genome Res. 11, 163–169. 10.1101/gr.157901 11156625 PMC311033

[B46] Nazarenko I. A. Bhatnagar S. K. Hohman R. J. (1997). A closed tube format for amplification and detection of DNA based on energy transfer. Nucleic Acids Res. 25, 2516–2521. 10.1093/nar/25.12.2516 9171107 PMC146748

[B47] Peatman E.. (2011). SNP genotyping platforms, in Next Generation Sequencing and Whole Genome Selection in Aquaculture, ed Liu Z. (Oxford: Blackwell Publishing), 123–132. 10.1002/9780470958964.ch8

[B48] Perkel J.. (2008). SNP genotyping: six technologies that keyed a revolution. Nat. Methods 5, 447–453. 10.1038/nmeth0508-447

[B49] Ragoussis J.. (2009). Genotyping technologies for genetic research. Annu. Rev. Genomics Hum. Genet. 10, 117–133. 10.1146/annurev-genom-082908-150116 19453250

[B50] Rasheed A. Hao Y. Xia X. Khan A. Xu Y. Varshney R. K. . (2017). Crop breeding chips and genotyping platforms: progress, challenges, and perspectives. Mol. Plant. 10, 1047–1064. 10.1016/j.molp.2017.06.008 28669791

[B51] Rasheed A. Wen W. Gao F. Zhai S. Jin H. Liu J. . (2016). Development and validation of KASP assays for genes underpinning key economic traits in bread wheat. Theor. Appl. Genet. 129, 1843–1860. 10.1007/s00122-016-2743-x 27306516

[B52] Rickert A. M. Borodina T. A. Kuhn E. J. Lehrach H. Sperling S. (2004). Refinement of single-nucleotide polymorphism genotyping methods on human genomic DNA: amplifluor allele-specific polymerase chain reaction versus ligation detection reaction-TaqMan. Anal Biochem. 330, 288–297. 10.1016/j.ab.2004.03.035 15203335

[B53] Rosas J. E. Bonnecarrère V. Pérez de Vida F. (2014). One-step, codominant detection of imidazolinone resistance mutations in weedy rice (*Oryza sativa* L.). Electron. J. Biotechnol. 17, 95–101. 10.1016/j.ejbt.2014.02.003

[B54] Ryu J. Kim W. J. Im J. Kang K. W. Kim S. H. Jo Y. D. . (2019). Single nucleotide polymorphism (SNP) discovery through genotyping-by-sequencing (GBS) and genetic characterization of *Dendrobium* mutants and cultivars. Sci. Hortic. 244, 225–233. 10.1016/j.scienta.2018.09.053

[B55] Ryu J. Kim W. J. Im J. Kim S. H. Lee K. S. Jo H. J. . (2018). Genotyping-by-sequencing based single nucleotide polymorphisms enabled Kompetitive Allele Specific PCR marker development in mutant *Rubus* genotypes. Electron. J. Biotechnol. 35, 57–62. 10.1016/j.ejbt.2018.08.001

[B56] SantaLucia J. J.. (1998). A unified view of polymer, dumbbell, and oligonucleotide DNA nearest-neighbour thermodynamics. Proc. Natl. Acad. Sci. USA 95, 1460–1465. 10.1073/pnas.95.4.1460 9465037 PMC19045

[B57] Schena L. Hughes K. J. D. Cooke D. E. L. (2006). Detection and quantification of *Phytophthora ramorum, P. kernoviae, P. citricola* and *P. quercina* in symptomatic leaves by multiplex real-time *PCR*. Mol. Plant Pathol. 7, 365–379. 10.1111/j.1364-3703.2006.00345.x 20507453

[B58] Schramm C. Shavrukov Y. Anderson P. Kurishbaev A. Jatayev S. (2019). Development of Single nucleotide polymorphism (SNP) markers for cereal breeding and crop research: current methods and future prospects, in Advances in Breeding Techniques for Cereal Crops, eds Ordon F. Friedt W. (Cambridge: BD Publishing), 327–362. 10.19103/AS.2019.0051.16

[B59] Shavrukov Y. Gupta N. K. Miyazaki J. Baho M. N. Chalmers K. J. Tester M. . (2010). *HvNax3*—a locus controlling shoot sodium exclusion derived from wild barley (*Hordeum vulgare* ssp. spontaneum). Funct. Integr. Genomics 10, 277–291. 10.1007/s10142-009-0153-8 20076983

[B60] Shavrukov Y. Zhumalin A. Serikbay D. Botayeva M. Otemisova A. Absattarova A. . (2016). Expression level of the DREB2-type gene, identified with Amplifluor SNP markers, correlates with performance, and tolerance to dehydration in bread wheat cultivars from Northern Kazakhstan. Front. Plant Sci. 7:1736. 10.3389/fpls.2016.01736 27917186 PMC5114286

[B61] Solinas A. Brown L. J. McKeen C. Mellor J. M. Nicol J. Thelwell N. . (2001). Duplex Scorpion primers in SNP analysis and FRET applications. Nucleic Acids Res. 29:e96. 10.1093/nar/29.20.e96 11600715 PMC60224

[B62] Stadhouders R. Pas S. D. Anber J. Voermans J. Mes T. H. M. Schutten M. (2010). The effect of primer-template mismatches on the detection and quantification of nucleic acids using the 5′ nuclease assay. J. Mol. Diagn. 12, 109–117. 10.2353/jmoldx.2010.090035 19948821 PMC2797725

[B63] Sweetman C. Khassanova G. Miller T. K. Booth N. J. Kurishbayev A. Jatayev S. . (2020). Salt-induced expression of intracellular vesicle tracking genes, *CaRab-*GTP, and their association with Na^+^ accumulation in leaves of chickpea (*Cicer arietinum* L.). BMC Plant Biol. 20:183. 10.1186/s12870-020-02331-5 33050887 PMC7557026

[B64] Thelwell N. Millington S. Solinas A. Booth J. Brown T. (2000). Mode of action and application of Scorpion primers to mutataon detection. Nucleic Acids Res. 28, 3752–3761. 10.1093/nar/28.19.3752 11000267 PMC110766

[B65] Thomson M. J.. (2014). High-throughput SNP genotyping to accelerate crop improvement. Plant Breed. Biotech. 2, 195–212. 10.9787/PBB.2014.2.3.19529356847

[B66] Udoh L. I. Obaseojei W. P. Uzoebo C. (2020). Single nucleotide polymorphisms: a modern tool to screen plants for desirable traits, in Plant Breeding—Current and Future Views, ed Abdurakhmonov I. Y. (London: InTech Open). 10.5772/intechopen.94935

[B67] Wang B. Tan H. W. Fang W. Meinhardt L. W. Mischke S. Matsumoto T. . (2015). Developing single nucleotide polymorphism (SNP) markers from transcriptome sequences for identification of longan (*Dimocarpus longan*) germplasm. Hort. Res. 2:14065. 10.1038/hortres.2014.65 26504559 PMC4595986

[B68] Weck S. Peterseil V. Mayer H. K. Hochegger R. (2021). Development and validation of a real-time PCR assay to detect *Cannabis sativa* in food. Sci. Rep. 11:4748. 10.1038/s41598-021-83908-4 33637785 PMC7910487

[B69] Wu J. H. Hong P. Y. Liu W. T. (2009). Quantitative effects of position and type of single mismatch on single base primer extension. J. Microbiol. Methods 77, 267–275. 10.1016/j.mimet.2009.03.001 19285527

[B70] Wu Y. Li M. He Z. Dreisigacker S. Wen W. Jin H. . (2020). Development and validation of high-throughput and low-cost STARP assays for genes underpinning economically important traits in wheat. Theor. Appl. Genet. 133, 2431–2450. 10.1007/s00122-020-03609-w 32451598

[B71] You Q. Yang X. Peng Z. Xu L. Wang J. (2018). Development and applications of a high throughput genotyping tool for polyploid crops: Single nucleotide polymorphism (SNP) array. Front. Plant Sci. 9:104. 10.3389/fpls.2018.00104 29467780 PMC5808122

[B72] Zhao Y. Wang K. Wang W. L. Yin T. T. Dong W. Q. Xu C. J. (2019). A high-throughput SNP discovery strategy for RNA-seq data. BMC Genomics 20:160. 10.1186/s12864-019-5533-4 30813897 PMC6391812

[B73] Zhu Z. Sun B. Lei J. (2021). Specific-locus amplified fragment sequencing (SLAF-Seq) as high-throughput SNP genotyping methods, in Crop Breeding. Methods in Molecular Biology, Vol. 2264, ed Tripodi P. (New York: Humana), 75–87. 10.1007/978-1-0716-1201-9_6 33263904

[B74] Zlotina M. M. Kovaleva O. N. Loskutov I. G. Potokina E. K. (2013). The use of allele-specific markers of the *Ppd* and *Vrn* genes for predicting growing-season duration in barley cultivars. Russ. J. Genet.: Appl. Res. 3, 254–264. 10.1134/S2079059713040114

[B75] Zotova L. Kurishbayev A. Jatayev S. Goncharov N. P. Shamambayeva N. Kashapov A. . (2019). The general transcription repressor *TaDr1* is co-expressed with *TaVrn1* and *TaFT1* in bread wheat under drought. Front. Genet. 10:63. 10.3389/fgene.2019.00063 30800144 PMC6375888

[B76] Zotova L. Kurishbayev A. Jatayev S. Khassanova G. Zhubatkanov A. Serikbay D. . (2018). Genes encoding transcription factors TaDREB5 and TaNFYC-A7 are differentially expressed in leaves of bread wheat in response to drought, dehydration and ABA. Front. Plant Sci. 9:1441. 10.3389/fpls.2018.01441 30319682 PMC6171087

[B77] Zotova L. Shamambaeva N. Lethola K. Alharthi B. Vavilova V. Smolenskaya S. E. . (2020). *TaDrAp1* and *TaDrAp2*, partner genes of a transcription repressor, coordinate plant development and drought tolerance in spelt and bread wheat. Int. J. Mol. Sci. 21:8296. 10.3390/ijms21218296 33167455 PMC7663959

