# Supplementary material for: Modified “Allele-Specific qPCR” Method for SNP Genotyping Based on FRET
Source: Front Plant Sci. 2022 Jan 10;12:747886. doi: 10.3389/fpls.2021.747886 (PMC8784781; doi:10.3389/fpls.2021.747886)
Supplement: Supplementary file 2 [file Data_Sheet_1.PDF]

Supplementary Material

Supplementary material S1

Figures from web-sites of Integrated DNA Technologies for rhAmp (A), and from LGC Genomics for KASP (B), showing structures of ASPs and UPs

A

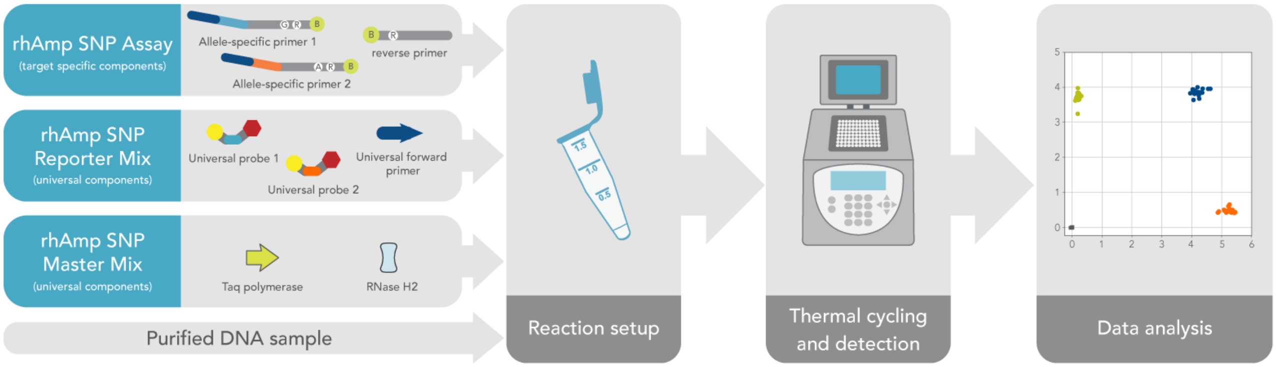

[https://sg.idtdna.com/pages/products/qpcr-and-pcr/genotyping/rhamp-snp-genotyping]

B

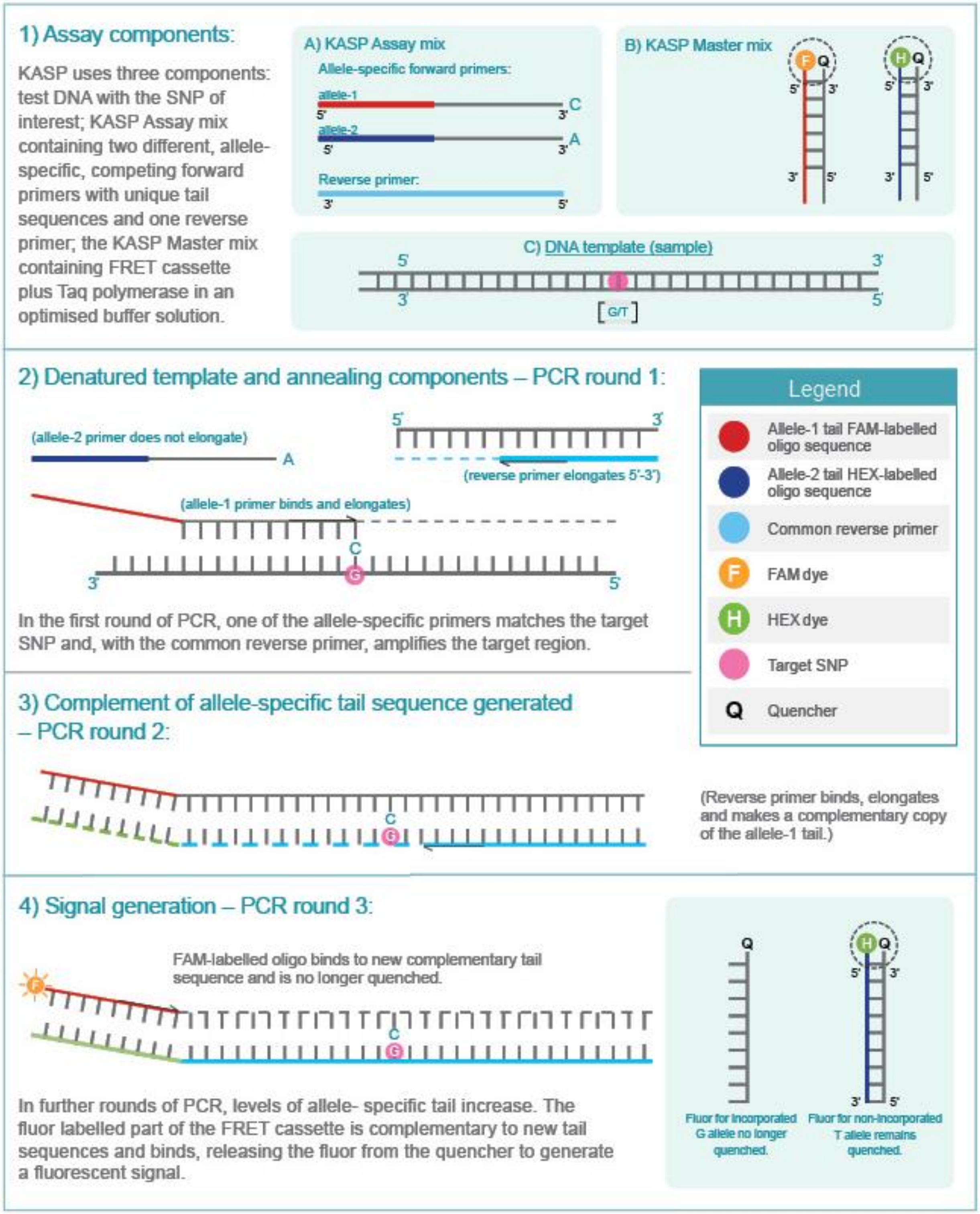

[http://info.biosearchtech.com/agrigenomics-pcr-based-kasp-genotyping?utm_campaign=SO-KASP-Targeted&utm_medium=cpc&utm_source=googleads&gclid=EAIaIQobChMI1oXLhYWN8wIV-ZlmAh09qAEAEAAYASAAEgLJPvD_BwE]

Supplementary material S2

Target gene *HvSAP16* in barley: annotated sequence, primer design for sequencing and supporting information, conditions of PCR, purification of PCR products and sequencing

MLOC_52196 = AK360983

Zinc-finger AN1 and C2H2 domain-containing stress-associated protein 16

The contig was identified from barley cv. Bowman reference genome using IPK database (https://galaxy-web.ipk-gatersleben.de). The designed primer positions are highlighted in grey. The identified SNP is shown in yellow. 'Start' and 'Stop' codons in the target gene *HvSAP16* are indicated in green and red, respectively. Design and supporting information for primers is present below.

>Bowman_contig_183489 CAJX010179134 carma=2HS

```
CCCCCCCCCCCCCCTCGATTCTCATCCCAACATGCAGTCCCTACATATATTAATCTGGCGATGCTTACCTGAAGTATCTGT
TTAAAAACTATAAAGTGTGGCAGAATTTTGAATACTGACTAAATATAAGAGATGCAAAATCCTTTCTTTTCGCTCAAAATG
TATTCAATCCTTCTGGACTTCCTGACTTTCTTAATATGAAAATAAGATTGCACATTTGTACTGAGGATGGTAGTTATTTAT
AGCGATTAGTAAAGATTGCACGCGATTAGAGAAGCTTGTCCAACATCGTCTGANNNNNTTTGGGCATATTCAGCGCAGGCC
TCCAGAAGCCCCAGTGCATAGCGGACGGCTAAAGCATTGTTGATAATGTCAAGAGAGGCCAGGGTAGACCGAATTTGACAT
GAGAAGAGTCCGTAAAGAGAGATCTAAAGGATTGGAGCATCACCAAAGAACTAGCCATGGACTGGGCTGCGTGGAAGCTTG
CTATCCATGTGCCAGAACCATGAGTTGGTCGCGAGATCTAATGGGTTTCACCTCTAGCCTACCCNNNNNNNNNNNNNNNTA
AAGGCTTTGTTGTTGTTGTTGTGGTAGTGAAGAGTGAAGTTTTTTGCTGAGTTAACTGTATTCAACTTTTTTTTCACAGAA
TATGTAGTATTTTATTCTTATGGAAATTGATTCCATTTTACCTTCTGAAACTATTTTGTCTTTTGAAATGCACTGGCACCT
TAATTATAATAGTTCTGTCATCACCAAGTGCAGCCAGCCATTGTGCTAATATGGCTATTGTATGATTGTTCAGGTCTTTTG
CCTTCAGCACCGAAGTTATACATCACATAAATGTCCAAAGGCTAATAATAAGGATGTCACTGTTGTTGTGTGCCCATTATG
TGCTAAAGGAGTTCGCCTCAATCCAAATGAAGATGCAAACATCACCTGGGAGTCTCATGTTAATACCGACTGCGATCCATC
AAATTACCAGAGAGCAACAAAGAAAAAGAAATGCCCAGTTCCTGGGTGCAGAGAAGCACTGACATTTTCAAACACAATCAT
GTGCAAAGATTGCAGCAAAGAGCACTGCCTCAAGCACAGATTTGGTCCGGATCACAAGTGTCCTGGCCCAAGAAAACCGGA
ACCTACCTTCCCCTTTTCCAACATGCTAAGGAGAAGTCAGAAAGTTGAGCCGCGCGCCAACAGCAGTAGCAGTAGTTCTTC
CTGGTGGAGCTCCAGCCTTTTGAATGCAGCATCAAGTTTCAGATCATCGGCAGAAGCAGGAATGCAAAAATTGAGCATTGC
CACCAGCGAAGCCGTCCAGAAGGCTAAGGATGGGGTCACCCCGAGCAGCAGTGGCAGTGGCAGTGGCCTCGTGGAGCAATG
TGTTCAGTGTCCAGCAAGGTTTTCCACTGTTGGGGCCCTAATCGAGCACGCCGAGAAGTCCCATGGGAACAACTCGCAACC
AAGCCGTGGTAAAGTGACGATCGATGCTTGCCCCAAATGTAGCAAGGGATTTCAAGATCCGGTGTTACTAGTGGAGCATGT
TGAGAGGGAACATGGAGGGACTTCAAAAGCATAAACCTGCCCATGAGTCATTTCTCATGAGTATGTTCTCATGAGTATGTT
ATATGCCTGATTGCTGTGTAATTTACTCCTCATTTACTCGATTTGATTGTACTTTAGTGAGCTCTGGGCTCGAACTGTGCA
CATGAAAGGAAGTCTTATCTCAAGGCAAGCCATTG
```

Primers for sequencing:

HvSAP16-seq-F: 5'-**ATCCCAACATGCAGTCCCTACA** 22 bp, 50%GC, Basic Tm=54.7C

HvSAP16-seq-R: 5'-**ATTAGCACAATGGCTGGCTGCA** 22 bp, 50%GC, Basic Tm=58.4C

(Rev.Com): TGCAGCCAGCCATTGTGCTAAT

Amplicon size: 756 bp

PCR conditions for sequencing:

PCR was conducted in individual tubes with total reaction volume of 30 µL. Template DNA was extracted using phenol-chloroform method and adjusted for 20 ng/µL. The final concentration of the used components following the manufacturer's protocol was as follows: (1) NEBiolab Q5 Reaction Buffer ($MgCl_2$ included) = × 1; (2) dNTP = 0.2 mM; (3) F and R primers = 0.5 µM; (4) Q5 High-Fidelity DNA polymerase (NEBiolab, USA) = 0.02 units; (5) Sterile water = To adjust a volume. Template DNA (2.5 ng, final conc.) was added to Master-mix loaded in each tube.

PCR amplification, purification of PCR products and sequencing:
Amplification was carried out in a Thermal MyCycler (BioRad, USA) with the following program: Initial denaturation at 98°C for 2 min; 35 cycles of 98°C for 10 s, 58°C for 10 s, and 72°C for 40 s; and with a final extension at 72°C for 3 min. Small volume (5 µL) of PCR products were checked after separation by electrophoresis in 1.0% agarose gels stained with GelRed (Biotium, USA) with a 1 Kb DNA Ladder (Bioline, USA), and visualized under UV light using a GelDoc system (BioRad, USA). The rest of PCR products (25 µL) were purified using PCR Purification Kit (Bioline, USA), checked for concentration and quality control using NanoDrop (ThermiFisher Scientific, USA) and submitted for sequencing in both directions (separately, with forward and reverse primers) using ABI-BigDye sequencing service at AGRF (Adelaide, Australia).

Supplementary Material S3

Sequences of SNP, primer design and their characteristics of the proposed ASQ method in *HvSAP8* in barley

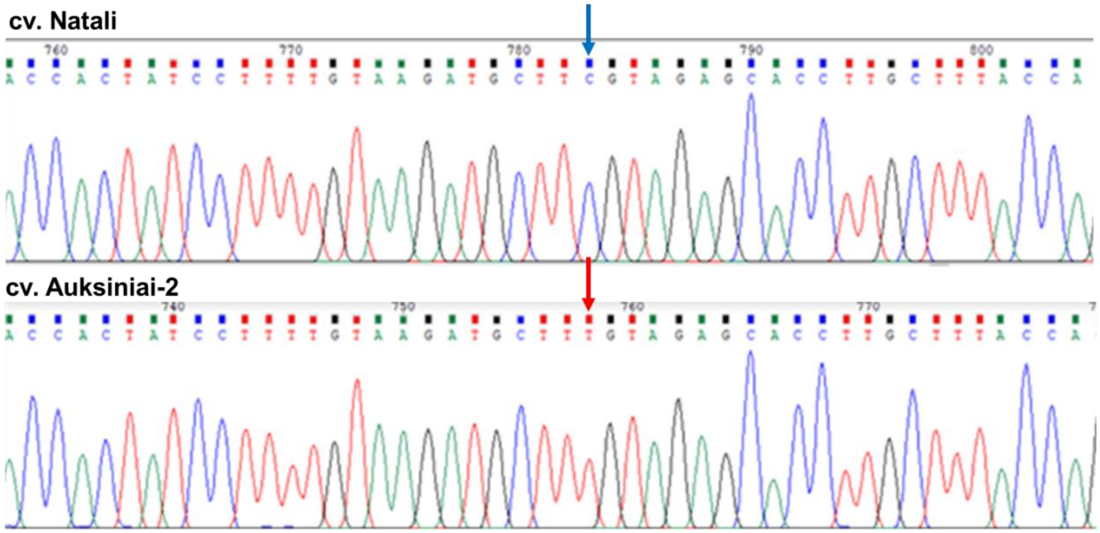

Supplementary Figure S3-1. Fragments of *HvSAP8* gene sequences in barley cvs. Natali and Auksiniai-2 with the identified SNPs, designated by blue and red arrows for 'C' and 'T' nucleotides, respectively.

```
AAATTTCCCCTGCTCTGCTCCGATGCCGGTAGGTGATTAGGCCGCTGTTGGCTAATTATAAGC
CAGGCAGGTTGCATCGTTTAGTGCTGGTGCCTTCAGCTCTGTGTTTGACCAAGCAGCCACCAC
TATCCTTTTGTAAGATGCTT[Y]GTAGAGCACCTTGCTTTACCAGAGTTGGATTCAGTAGGCT
AAAACATACTGTRTGTTTGAAGATTATGCCATGTTCATCTCTAAATTGTTAAAGCCGCAGTGA
```

[Y] = [C/T]

<u>Primers:</u>

HvSAP8-F7: 5'-ccagctgaacggt***TCGACC***GGTAAAGCAAGGTGCTCTACGA-3'

HvSAP8-F8: 5'-ccagctgaacggt***ACCTGC***TGGTAAAGCAAGGTGCTCTACAA-3'

HvSAP8-R7-8: 5'-CGTTTAGTGCTGGTGCCTTCA-3'

Supplementary Figure S3-2. Development of allele-specific primers for SNP genotyping of *HvSAP8* in barley. The explanation if similar to those in the legend of Figure 2 in the main text and as follows. Forward and reverse primers are highlighted in green and yellow, respectively. The SNP is designated as '[Y]' in red, and may be either 'C' or 'T' nucleotides. The design of two forward primers in the reverse-complement sequence is shown in the lower part of the Figure. The uncoloured 19-bp tag containing a 13-bp identical sequence (in lower case letters) and 6-bp unique barcode fragment (in Capital Italic letters) is shown. The common reverse primer (in yellow) is the same as present in the gene sequence.

Supplementary Table S3-3. Sequences and characteristics of allele-specific primers for SNP genotyping of *HvSAP8* in barley.

Primer ID	Sequence (5′-3′)	T_m (°C)[1]	T_m (°C)[2]	dG (kcal /mole)
HvSAP8-F7	ccagctgaacggt***TCGACC-***GGTAAAGCAAGGTGCTCTAC**G**A	64.7	56.4	-27.4
HvSAP8-F8	ccagctgaacggt***ACCTGC-***TGGTAAAGCAAGGTGCTCTAC**A**A	64.1	55.8	-27.9
HvSAP8-R7-8	CGTTTAGTGCTGGTGCCTTCA	65.6	57.4	-27.2

[1] Temperature melting (T_m) was calculated for ASP oligonucleotide sequences without tail based on concentration for 250 nM, 50 mM K^+ and with 3 mM Mg^{2+};

[2] Temperature melting (T_m) calculated the same as in ([1]) but in the absence of Mg^{2+};

Supplementary material S4

Design of allele-specific primers (ASPs) and universal probes (UPs), and PCR conditions for SNP genotyping in barley target gene *HvSAP16* using Amplifluor-like and KASP methods

The fragment sequence in the promoter region of the *HvSAP16* gene in barley (*Hordeum vulgare* L.) with targeted SNP and developed ASPs. Forward and reverse primers are highlighted in pink and yellow colour, respectively. The SNP is designated as '[W]' in red, and may be either 'A' or 'T' nucleotides. The design of Amplifluor-like primers is shown below, with the uncoloured 21-bp tag containing a 6-bp identical part (in lower case letters) and 15-bp unique barcode fragment (in Capital Italic letters). The Universal probes used in Amplifluor-like SNP genotyping (Uni-1 and Uni-2) shown below have FAM or HEX/VIC fluorophores in 5'-end, quencher (BHQ1) in the middle of the probe and the tags in 3'-end, identical to those in ASP.

```
TCCTTTCTTTTCGCTCAAAATGTATTCAATCCTTCTGGACTTCCTGACTTTCTTAATATGAAA
ATAAGATTGCACATTTGTACTGAGGATGGTAGTTATTTATAGCGATTAGTAAAGATTGCACGC
GATTAG[W]GAAGCTTGTCCAACATCGTCTGAGATGGTTTGGGCATATTCAGCGCAGGCCTCC
AGAAGCCCCAGTGCATAGCGGACGGCTAAAGCATTGTTGATAATGTCAAGAGAGGCCAGGGTA
[W] = [A/T]
```

HvSAP16-Amp-F1: 5'-**AGACGATGTTGGACAAGCTTCTC** 23 bp; GC=48%; Tm=56.2C
(RevCom): **GAGAAGCTTGTCCAACATCGTCT**

HvSAP16-Amp-F2: 5'-**AGACGATGTTGGACAAGCTTCAC** 23 bp; GC=48%; Tm=56.0C
(RevCom): **GTGAAGCTTGTCCAACATCGTCT**

HvSAP16-Amp-R: **GCACATTTGTACTGAGGATGGTAG** 24 bp; GC=46%, Tm=54.6C

Amplicon size: 83 bp.

Amplifluor Allele-specific primers:
HvSAP16-Amp-F1: gaaggt***GACCAAGTTCATGCT*****AGACGATGTTGGACAAGCTTCTC**
HvSAP16-Amp-F2: gaaggt***CGGAGTCAACGGATT*****AGACGATGTTGGACAAGCTTCAC**
HvSAP16-Amp-R: **GCACATTTGTACTGAGGATGGTAG**

Amplifluor Universal probes:
Uni-1: 5'-FAM-AGCGATGCGTTCGAGCATCGC(T*-BHQ1)gaaggt***GACCAAGTTCATGCT***
Uni-2: 5'-HEX-AGGACGCTGAGATGCGTCC(T*-BHQ1)gaaggt***CGGAGTCAACGGATT***

PCR conditions used for Amplifluor-like SNP genotyping:
Each reaction with a total volume of 10 µl contained: 2 µL of template DNA adjusted to 20 ng/µL, 1 µL of the two fluorescently-labelled Universal probes mix (0.125 µM each), 1 µL of allele-specific primer mix (0.075 µM of each of two forward primers and 0.39 µM of the common reverse primer), 2 µL of clear 5×Go-Taq Master-mix (Promega, USA) and 0.08 µL of Go-Taq DNA polymerase (5 units / µL), with the following final concentration of components: 1.5 mM $MgCl_2$, 0.2 mM of dNTP and 0.04 units of Go-Taq DNA polymerase (Promega, USA).

Amplification conditions and SNP genotyping using Amplifluor-like method:
Amplifluor-like SNP analysis was carried out using a QuantStudio-7 Real-Time PCR instrument (ThermoFisher Scientific, USA) and CFX96 Real-Time PCR Detection System (BioRad, USA). PCR was conducted using adjusted program: Initial denaturation, 94°C, 2 min; 20 ‘doubled’ cycles of 94°C for 10 s, 55°C for 1 min, 94°C for 10 s, 50°C for 1 min; with recording of allele-specific fluorescence after each cycle. Genotyping by SNP calling was determined automatically by the instrument software, but each SNP result was also checked manually using amplification curves and final allele discrimination. The experiments were repeated twice (two technical replicates), confirming the confidence of SNP calls.

PCR conditions used for KASP genotyping:
Each reaction with a total volume of 5 µL contained: 0.5 µL of template DNA adjusted to 20 ng/µL, 0.5 µL of Amplifluor-like allele-specific primer mix, the same as described above (0.075 µM of each of two forward primers and 0.39 µM of the common reverse primer), and 4 µL of 1×KASP Master-Mix with fluorescently-labelled probes, other components and DNA polymerase commercially produced by LGC Genomics, UK.

Amplification conditions and SNP genotyping using KASP method:
KASP analysis was carried out using the same QuantStudio-7 Real-Time PCR instrument (ThermoFisher Scientific, USA) and CFX96 Real-Time PCR Detection System (BioRad, USA). PCR was conducted using adjusted program: Initial denaturation, 94°C, 15 min; 10 ‘touch-down’ cycles including 94°C for 20 s, 65°C for 1 min, with following reducing annealing temperature for 1°C per cycle; 30-33 cycles including 94°C for 20 s, 55°C for 1 min; with recording of allele-specific fluorescence after each cycle. Genotyping by SNP calling was determined automatically by the instrument software, but each SNP result was also checked manually using amplification curves and final allele discrimination. The experiments were repeated twice (two technical replicates), confirming the confidence of SNP calls.

[Supplementary material S5 is present in a separate Excel-file]

Supplementary material S6

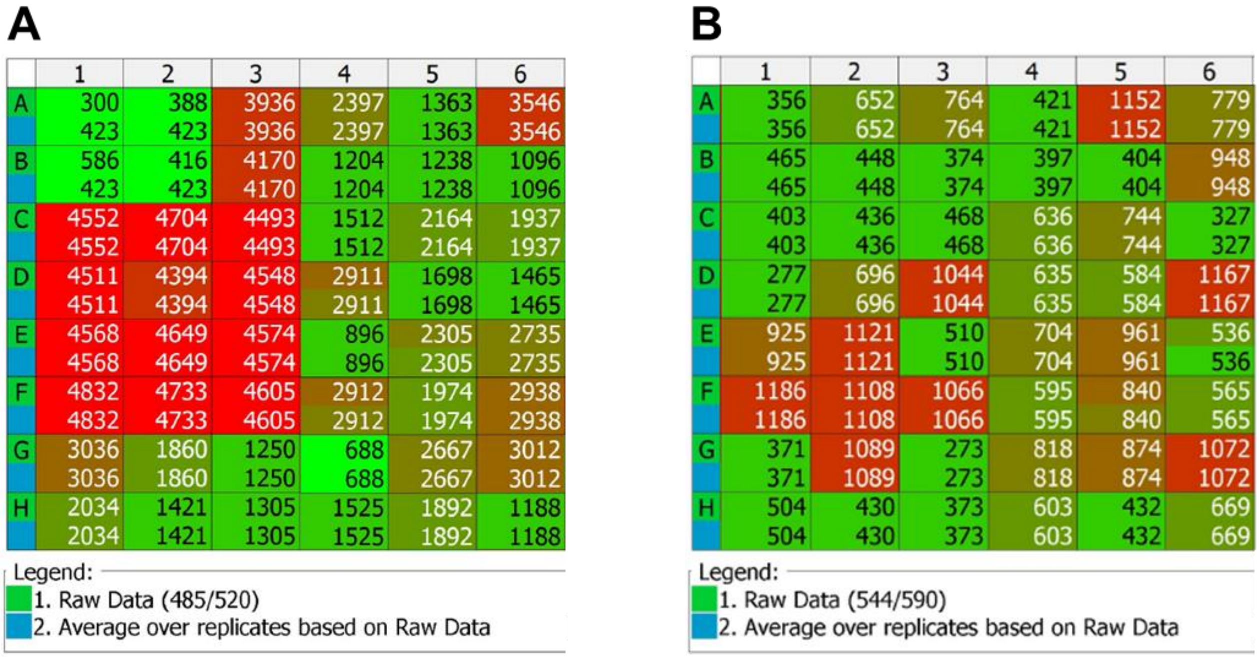

A

	1	2	3	4	5	6
A	300	388	3936	2397	1363	3546
	423	423	3936	2397	1363	3546
B	586	416	4170	1204	1238	1096
	423	423	4170	1204	1238	1096
C	4552	4704	4493	1512	2164	1937
	4552	4704	4493	1512	2164	1937
D	4511	4394	4548	2911	1698	1465
	4511	4394	4548	2911	1698	1465
E	4568	4649	4574	896	2305	2735
	4568	4649	4574	896	2305	2735
F	4832	4733	4605	2912	1974	2938
	4832	4733	4605	2912	1974	2938
G	3036	1860	1250	688	2667	3012
	3036	1860	1250	688	2667	3012
H	2034	1421	1305	1525	1892	1188
	2034	1421	1305	1525	1892	1188

B

	1	2	3	4	5	6
A	356	652	764	421	1152	779
	356	652	764	421	1152	779
B	465	448	374	397	404	948
	465	448	374	397	404	948
C	403	436	468	636	744	327
	403	436	468	636	744	327
D	277	696	1044	635	584	1167
	277	696	1044	635	584	1167
E	925	1121	510	704	961	536
	925	1121	510	704	961	536
F	1186	1108	1066	595	840	565
	1186	1108	1066	595	840	565
G	371	1089	273	818	874	1072
	371	1089	273	818	874	1072
H	504	430	373	603	432	669
	504	430	373	603	432	669

Supplementary Figure S6. Example of results in graphical form of end-point fluorescence units of FAM **(A)** and HEX **(B)** using a Microplate Reader. As explained in the Figure Footnote, each square has two data: raw data (1) and the average of three technical replicates (2). Low, medium and high levels of fluorescence emission/absorption are also highlighted in green, brown and red, respectively.

Supplementary material S7

Estimated cost per SNP and expenses for reagents used in different methods for SNP genotyping

Each PCR cocktail-mix contains only three major components: (1) DNA template; (2) set of allele-specific primers ASPs (two forward and one common reverse); and (3) Master-mix (KASP and Amplifluor) or set of UPs with dyes and Uni-Q (our proposed ASQ method).

The first two components (DNA and ASPs) are common for all these methods. ASPs without dyes or quenchers are equally required for KASP, Amplifluor and ASQ methods, with practically the same cost varying by plus/minus 1-5 nucleotides in the length. The only component No 3 was different. Therefore, the universal molecular probes (UPs) with two fluorophores (UP-1 and UP-2) and universal probe with a quencher (Uni-Q) were estimated as the most expensive components as ordered separately. Commercial products for SNP genotyping system (Millipore-Merck) and KASP Master-mix (LGC Genomics) were estimated as price-list request. The final calculation of the cost per SNP in one reaction is present.

The Proposed ASQ method: estimated costs of FRET (UP-1, UP-2 and Uni-Q) and ASP components in three companies:
(1) **DNA Synthesis** (Moscow, Russia): **FRET and ASP components** for 10,000 x 10 µL reactions: 2,100 + 2,400 + 3,400 +1,100 + 1,100 + 456 = RRub 10,556 = USD 147, equivalent to USD **0.0147** per SNP in one reaction [Based on price-list provided at: https://oligos.ru/price.html].

(2) **Sigma-Aldrich-Merck** (Sydney, Australia): **FRET and ASP components** for 10,000 x 10 µL reactions: 75 + 75 + 250 + 10 + 10 + 5 = AUD 425 = USD 312, equivalent to USD **0.0312** per SNP in one reaction [Based on pre-order of the products at: https://www.sigmaaldrich.com/AU/en].

(3) **Eurofins Genomics** (Ebensburg, Germany): **FRET and ASP components** for 10,000 x 10 µL reactions: 60 + 60 + 128 + 8 + 8 + 5 = Euro 269 = USD 309, equivalent to USD **0.0309** per SNP in one reaction [Based on pre-order of the products at: https://eurofinsgenomics.eu].

KASP and Amplifluor: estimated costs of commercial products and ASP components:
(4) **KASP** (LGC Genomics, UK), Catalog No: KBS-1016-002, KASP Master-mix for 5,000 x 10 µL reactions: USD 1,000 + ASP set: USD 18-25, equivalent to about USD **0.202** per SNP in one reaction [Based on pre-order of the products at: www.lgcgroup.com/genomics].

(5) **Amplifluor** SNP Genotyping System (Millipore-Merck, USA), Catalog No: S7908 and S7909 for 5,000 x 10 µL reactions: USD 3,000 + ASP set: USD 18-25, equivalent to about USD **0.602** per SNP in one reaction [https://www.merckmillipore.com].

We provided our information to best of our knowledge on the stage of the manuscript submission. We also state that prices can be variable and changed in future but this is behind of our control.
